# Relationships of Multiphase Structure with Flexibility and DC Insulation Performance in Impact-Resistant Polypropylene Copolymers

**DOI:** 10.3390/polym18192351

**Published:** 2026-09-27

**Authors:** Wenqin Zhu, Mi An, Jingsheng Zhou, Bin Du, Qiang Xu, Yingjie Zhang, Hongming Li, Weihuan Huang

**Affiliations:** 1 College of Mechanical and Electrical Engineering, Beijing University of Chemical Technology, Beijing 100029, China; 2 Petrochemical Research Institute of PetroChina, Beijing 102206, China; 3 CNPC Key Laboratory of Synthetic Resin and New Materials, Beijing 102206, China

**Keywords:** impact-resistant polypropylene copolymer, multiphase topology, interfacial coupling, space charge, DC breakdown, flexibility

## Abstract

Softening impact-resistant polypropylene (PP) copolymers (ICPs) may be accompanied by reduced breakdown performance; coordinating mechanical and electrical properties is closely related to crystalline-framework and rubber-rich-phase organization. Homopolymer PP matrix HICP and random copolymer PP matrix RICP formed the primary comparison, with high-phase-continuity CICP as a reference, through composition, fractionation, crystalline structure, morphology, viscoelasticity, and charge analyses. Relative to HICP, RICP matrix randomization extended crystallizability distributions toward lower temperatures, refined the crystalline framework, and expanded interphase regions. Tensile and flexural moduli decreased by 38.8% and 38.7%, respectively, while Weibull characteristic breakdown strength increased from 226.2 to 310.8 kV/mm. CICP, containing 23.3 wt% ethylene, comprised a continuous low-crystallinity/rubber-rich phase with dispersed PP crystalline domains. Its tensile modulus was 89.4 ± 4.9 MPa; it retained partial break in notched impact testing at −20 °C and a characteristic breakdown strength of 384.4 kV/mm. Thermally stimulated depolarization current, conduction, and space-charge results indicated that local trapping environments and multiphase transport pathways jointly regulate charge migration and accumulation, providing a charge-behavior basis for maintaining high insulation performance as flexibility increases. The relationships linking molecular sequences, crystalline organization, phase connectivity, and mechanical–electrical responses provide a basis for designing multiphase PP-based DC cable insulation.

## 1. Introduction

High-voltage direct-current (HVDC) cable insulation requires electrical reliability, thermal stability, processability, and recyclability. Crosslinked polyethylene has an established application base, but its crosslinked network limits melt recycling, and removing crosslinking by-products adds manufacturing steps [1,2]. Polypropylene (PP) combines thermoplastic processability, a relatively high melting temperature, and good dielectric properties, providing a material basis for recyclable cable insulation. However, its high rigidity and low-temperature brittleness make flexibility an important consideration in practical applications [1,3].

Regulating copolymer sequences and blending with elastomers are common approaches to improving PP flexibility. Random ethylene units alter the length and distribution of crystallizable propylene sequences, with corresponding changes in crystallinity, crystallization kinetics, and stiffness. Elastomer blending improves deformability by adjusting the flexible-phase fraction and its dispersion [3,4]. However, softening and improved insulation do not necessarily occur together. For example, blending PP with propylene-based or ethylene-based elastomers substantially improved deformability, whereas DC breakdown strength decreased with increasing elastomer content, to different extents in the two blend systems [5]. Maintaining high-field insulation performance while reducing rigidity is therefore a key challenge in designing flexible PP-based cable insulation.

Impact-resistant polypropylene copolymers (ICPs) are produced by sequential in-reactor polymerization, forming a crystalline PP matrix and ethylene–propylene rubber (EPR)-rich components. They also contain copolymer chain populations with different crystallizabilities, allowing the flexible components and their multiphase organization to be regulated. In cable-insulation research, Yang et al. reported that an ICP containing 15% rubber phase had a lower tensile modulus and higher AC breakdown strength than homopolymer PP. Increasing the rubber-phase content to 30% reduced the characteristic AC breakdown strength from 154.9 to 117.8 kV/mm, below the 143.4 kV/mm of homopolymer PP [6]. In that system, further increasing the rubber-phase fraction changed an improvement in breakdown performance into a marked deterioration. Increasing the flexible-component content alone is therefore insufficient to coordinate mechanical and electrical performance. The structural conditions required to sustain high-field insulation at high flexible-component fractions must also be identified.

In ICPs, the crystallizability and phase partitioning of different chain populations provide the compositional basis for connections between crystalline supports and flexible regions. Fractionation and recombination studies show that intermediate-crystallizability components participate in compatibilization and connections between PP crystalline domains and rubber-rich regions, changing phase separation and viscoelastic responses [7]. Differences in copolymer sequences and interphase coupling also alter the internal structure of the rubber phase, producing different stiffness–toughness balances in materials of similar composition [8]. These organizational differences also affect interphase charge transport. Conductivity mismatch between soft and PP-rich phases can alter bulk space-charge accumulation and local field distortion [9]. Regulation of crystalline morphology and local trapping environments can affect charge trapping and breakdown [10,11]. Carrier migration and segmental motion both contribute to high-field failure [12]. It is therefore necessary to clarify how copolymer sequences regulate both load transfer and charge migration through changes in the crystalline framework and phase connectivity.

This study primarily compares homopolymer PP matrix HICP with random-copolymer-PP-matrix RICP, prepared using the same laboratory two-stage polymerization route, with matrix sequences regulated by introducing ethylene during the first stage. A commercial ICP with high phase continuity, designated CICP, is also used as a reference to examine structural and property responses when the low-crystallinity/rubber-rich phase is highly continuous. Nuclear magnetic resonance, molar-mass analysis, and cross-fractionation distinguish copolymer sequences and chain-population composition. Thermal analysis, X-ray scattering, microscopy, and viscoelastic analysis trace changes in crystalline supports and phase connectivity. Dielectric spectroscopy, high-field conduction, thermally stimulated depolarization current, and space-charge measurements examine the corresponding charge trapping, migration, and accumulation. Relating these structural and charge behaviors to mechanical and DC breakdown properties distinguishes the effects of matrix-sequence regulation and highly continuous phase organization on load transfer and charge transport. This comparison identifies structural conditions for maintaining high-field insulation during softening.

## 2. Materials and Methods

### 2.1. Materials

The high-phase-continuity reference CICP was commercial Hifax CA10A from LyondellBasell (Rotterdam, The Netherlands) and was used without further modification. Polymerization-grade propylene and ethylene had purities of at least 99.5% and 99.9%, respectively. Hydrogen and nitrogen both had purities of at least 99.999%. Table 1 lists the specifications, sources, and uses of the experimental reagents.

### 2.2. Two-Stage Polymerization of HICP and RICP

HICP and RICP were prepared at the Petrochemical Research Institute of PetroChina by two-stage polymerization in a 10 L stirred gas-phase reactor using an in-house-developed Ziegler–Natta catalyst system. Propylene and ethylene were purified through deoxygenation columns and molecular sieves before use. Catalyst components were weighed, diluted, and transferred under dry nitrogen. After three evacuation–nitrogen replacement cycles, the reactor was heated to 70 °C and stirred at 300 r/min. Triethylaluminum, 3.60 mmol (1.80 mL of a 2.0 mol/L solution in n-heptane), was added first. After 5 min of stirring, dicyclopentyldimethoxysilane (0.360 mmol; 1.44 mL of a 0.25 mol/L solution in n-heptane) was added. The MgCl_2_-supported TiCl_4_-type Ziegler–Natta catalyst was weighed to provide 0.030 mmol total Ti and predispersed in 2.0 mL anhydrous n-heptane. The suspension was injected through a metered feed port after the first-stage gas mixture had been established. The Al/Ti and Al/Si molar ratios were 120 and 10, respectively [13,14].

Propylene, ethylene, and hydrogen were metered separately using mass-flow controllers; all reaction pressures were absolute pressures. The first-stage temperature, total pressure, and gas-phase hydrogen/propylene molar ratio were 70 °C, 2.30 MPa, and 4.0 × 10^−3^, respectively. The gas mixture was first established according to the first-stage composition specified for each sample. Once temperature, pressure, and gas composition were stable, the catalyst suspension was injected, defining the start of the 60 min polymerization period. Propylene replenishment was controlled by pressure feedback. Ethylene and hydrogen replenishment rates were adjusted separately using compositions measured by online gas chromatography. Gas-phase ethylene/propylene composition is expressed as a molar ratio, whereas replenishment composition is expressed as a mass-flow ratio. Nitrogen and hydrogen were excluded from the normalized ethylene–propylene composition. Initial gas charges, cumulative feeds, and vented quantities were recorded separately for each stage. Net monomer conversion was calculated as total stage input minus total output and the increase in reactor inventory, including both free gas and monomer absorbed by the polymer [13,15,16].

#### 2.2.1. First-Stage Polymerization of HICP

Propylene was the only monomer in the first stage for HICP, giving a gas-phase ethylene/propylene molar ratio of 0:100. Hydrogen content was controlled using the hydrogen/propylene molar ratio specified above. Propylene was continuously supplied to maintain total pressure during polymerization, forming a homopolymer PP matrix. The first-stage product mass, m_1_, was determined from the net propylene conversion during this stage.

#### 2.2.2. First-Stage Polymerization of RICP

For RICP, the initial gas-phase ethylene/propylene molar ratio was set to 0.9:99.1 and maintained during polymerization. The initial ethylene replenishment mass-flow rate was 2/98 of the propylene mass-flow rate. Ethylene flow was subsequently adjusted using online gas-chromatography results to maintain the specified gas-phase ethylene/propylene molar ratio. Hydrogen replenishment was adjusted independently to maintain a gas-phase hydrogen/propylene molar ratio of 4.0 × 10^−3^. The first-stage temperature, total pressure, and polymerization time were identical to those for HICP. The sum of the net converted masses of ethylene and propylene was designated m_1_. The fraction of m_1_ attributable to net ethylene conversion defined the matrix ethylene mass fraction, approximately 2.0%.

#### 2.2.3. Second-Stage Copolymerization and Post-Treatment

At the end of the first stage, monomer and hydrogen replenishment was stopped. The temperature was reduced to 65 °C, and the total pressure was vented down to 0.10 MPa. Ethylene and propylene were then introduced to establish a gas-phase ethylene/propylene molar ratio of 39:61 and a hydrogen/(ethylene + propylene) molar ratio of 1.0 × 10^−3^, raising the total pressure to 1.80 MPa. The 45 min second-stage polymerization began once the specified temperature, pressure, and gas composition had been reached. The initial ethylene/propylene replenishment mass-flow ratio was 60:40. Propylene replenishment was subsequently adjusted by pressure feedback, while ethylene and hydrogen replenishment rates were corrected separately using online gas chromatography to maintain the specified gas composition. Catalytic sites retained in the first-stage polymer particles remained active during this stage; no additional primary catalyst, cocatalyst, or external electron donor was supplied. The total net converted mass of ethylene and propylene during stage switching and second-stage polymerization was designated m_2_. This was approximately 0.25 times m_1_, so the second-stage product accounted for approximately 20% of the total product mass. After the reaction, feeds were stopped, the reactor was cooled and depressurized, and high-purity nitrogen was passed through at 2.0 standard L/min for 30 min. The resulting powder was melt-pelletized at 190 °C and 80 r/min. Standard gas volumes refer to 0 °C and 101.325 kPa [15,16]. Figure 1 illustrates the first-stage matrix-sequence differences between HICP and RICP and the ethylene–propylene copolymer chain populations formed during the second stage.

### 2.3. Specimen Preparation

Pellets of the three materials were vacuum-dried at 80 °C for 8 h. Specimens were prepared in ambient air using a BP8170-B platen press (Dongguan Baopin Precision Instrument Co., Ltd., Dongguan, China). To prepare 0.20 mm films and 1.0 mm sheets, 2.0 and 10.0 g of pellets, respectively, were spread evenly in stainless-steel frame molds of the corresponding thickness, each with a 100 mm × 100 mm cavity. The 4.0 mm plates were prepared in a 180 mm × 60 mm frame mold with 43.0 g per mold. The material was preheated at 200 °C without pressure for 5 min [17] and compression-molded at 15 MPa for 5 min, with one brief depressurization during this period to release gas. Pressure was then maintained while circulating water cooled the material to room temperature at an average rate of 15 °C/min. After demolding and removal of flash, specimens were cut from the central plate region as required for each test. All specimens were conditioned for 48 h at 23 °C and 50% relative humidity.

Films approximately 0.20 mm thick were used for broadband dielectric spectroscopy, high-field conduction, thermally stimulated depolarization current (TSDC), pulsed electroacoustic (PEA), DC breakdown, and haze measurements. The 1.0 mm sheets were used for wide-angle/small-angle X-ray scattering (WAXS/SAXS), dynamic mechanical analysis (DMA), and dynamic rheology; 25 mm-diameter disks were cut for rheology. Tensile, flexural, and impact specimens were machined from the 4.0 mm plates.

Scanning electron microscopy (SEM) was used to examine extrudates approximately 2 mm in diameter, produced in a melt mass-flow-rate instrument at 230 °C under a 2.16 kg load. The extrudates were cooled in liquid nitrogen for 1 min and cryofractured. HICP and RICP fracture surfaces were etched in xylene at 80 °C for 8 h and then at 60 °C for 16 h. CICP surfaces were etched in xylene at room temperature for 1 h. The surfaces were rinsed successively with xylene and anhydrous ethanol, vacuum-dried at 40 °C for 12 h, and gold-coated. Polarized optical microscopy (POM) was performed on specimens approximately 30 μm thick, placed between glass slides [6].

Three independent measurement units (*n* = 3) were used for ^13^C nuclear magnetic resonance (NMR), high-temperature gel permeation chromatography with infrared detection (HT-GPC-IR), cross-fractionation chromatography (CFC), temperature-rising elution fractionation (TREF), differential scanning calorimetry (DSC), WAXS/SAXS, DMA, dynamic rheology, broadband dielectric spectroscopy, high-field conduction, TSDC, and PEA. For HICP and RICP, three independent polymerization batches were used for each material, with corresponding specimen preparation and measurement performed separately for each batch. For CICP, three independent sampling, preparation, and measurement procedures were performed using the same commercial batch. Each result was calculated independently before summarizing the mean ± sample standard deviation. Representative curves, spectra, and space-charge distributions correspond to results marked with superscript a in the tables.

### 2.4. Molecular Composition and Component Distributions

An 80 mg sample was placed in a high-temperature NMR tube and fully dissolved in 3.0 mL deuterated 1,2,4-trichlorobenzene at 120 °C. The ^13^C spectra were acquired on an AVANCE III 400 spectrometer (Bruker, Fällanden, Switzerland) using inverse-gated decoupling, a 90° pulse angle, a 10 s relaxation delay, and 4096 scans [18,19,20]. Peak assignments and monomer-content calculations followed Appendix A of the standard SH/T 1800–2016 [20]. S, T, and P denote methylene, methine, and methyl carbons, respectively; Greek-letter subscripts indicate their positions relative to neighboring methine carbons. The relative amounts of propylene and ethylene units, P′ and E′, were calculated from the integrated areas of the corresponding methylene resonance regions:

(1) $$P^{\prime }=S_{\alpha \alpha }+\frac{1}{2}(S_{\alpha \gamma }+S_{\alpha \delta })$$

(2) $$E^{\prime }=\frac{1}{2}[S_{\delta \delta }+S_{\alpha \delta }+S_{\alpha \gamma }+\frac{1}{2}(S_{\gamma \delta }+S_{\beta \delta }+S_{\alpha \delta })]$$

Here, each S term is the integrated area of the corresponding resonance region. The ethylene and propylene mole fractions are denoted E and P, respectively, with E + P = 1. The total ethylene mass fraction, *w_E_*, was obtained using the repeat-unit molar masses [20,21]:

(3) $$E=\frac{E^{\prime }}{P^{\prime }+E^{\prime }},\;\;P=\frac{P^{\prime }}{P^{\prime }+E^{\prime }}$$

(4) $$w_{E}=\frac{28.05E}{28.05E+42.08P}\times 100\%$$

Molar-mass distributions were measured using high-temperature gel permeation chromatography coupled with infrared detection (HT-GPC-IR; Polymer Char, Paterna, Spain). Samples were dissolved at 2.0 mg/mL in 1,2,4-trichlorobenzene containing 0.0125 wt% of the antioxidant butylated hydroxytoluene (BHT) at 150 °C for 90 min. Measurements were performed at 150 °C with a mobile-phase flow rate of 1.0 mL/min. Elution responses were recorded using an IR4 detector, and number-average and weight-average molar masses, ${\bar{M}}_{n}$ and ${\bar{M}}_{w}$, were obtained by universal calibration. Molar-mass dispersity, *Đ_M_*, was calculated as ${\bar{M}}_{w}$/${\bar{M}}_{n}$ for each measurement. The ${\bar{M}}_{w}$ difference between HICP and RICP was evaluated using a two-sided Welch t-test at a significance level of 0.05.

Cross-fractionation chromatography (CFC) and temperature-rising elution fractionation (TREF) were performed using a cross-fractionation chromatograph (Polymer Char, Paterna, Spain). Samples were dissolved at 2.0 mg/mL in 1,2,4-trichlorobenzene containing 0.0125 wt% BHT at 140 °C for 60 min; the injection volume was 500 μL. Samples were cooled from 140 to 30 °C at 1 °C/min and held for 30 min. TREF concentration responses were recorded during continuous heating from 30 to 140 °C at 1 °C/min [22]. For CFC, the fraction soluble at 30 °C was eluted first. The temperature was then increased in 5 °C steps from 35 to 140 °C, with heating between steps at 1 °C/min. Each step comprised a 5 min isothermal hold followed by elution at 1.0 mL/min and online size-exclusion chromatography (SEC); the analysis cycle per step was set to 45 min. The next temperature step began after complete elution and return to baseline. The SEC column was maintained at 150 °C. An IR4 concentration detector was coupled with an online capillary viscometer, and the molar-mass distribution of each fraction was obtained using the universal calibration described above. Fractions eluted at or below 35 °C were defined as the initial soluble fraction (SF). Its mass fraction and the elution-peak temperature of the principal PP-rich component were reported.

### 2.5. Thermal Properties and Crystalline Structure

Differential scanning calorimetry (DSC) was performed on a Q2000 instrument (TA Instruments, New Castle, DE, USA) under high-purity nitrogen. Samples of 5–8 mg were heated from room temperature to 200 °C at 10 °C/min, held for 5 min, cooled to 40 °C at 10 °C/min, and reheated to 200 °C at the same rate. The melting-peak temperature, *T_m_*, and crystallization-peak temperature, *T_c_*, were taken from the second-heating endotherm and cooling exotherm, respectively [23]. The melting enthalpy, Δ*H_m_*, normalized to total sample mass, was obtained by integrating the PP melting region of the second-heating curve. Because α-iPP was the principal crystalline phase in all three materials, the reference melting enthalpy of fully crystalline isotactic polypropylene, Δ*H_m_*^0^ = 207 J/g, was used consistently to calculate the apparent PP crystallinity, *X_c_*, on a whole-sample mass basis [24]:

(5) $$X_{c}=\frac{{\Delta H}_{m}}{{{\Delta H}_{m}}^{0}}\times 100\%$$

WAXS and SAXS were measured using a D8 Discover wide-angle/small-angle X-ray scattering instrument (Bruker, Karlsruhe, Germany) with Cu Kα radiation at a wavelength λ = 0.154 nm. WAXS scans covered 2θ = 10–30° with a 0.02° step and a 2°/min scan rate. Two-dimensional WAXS patterns were collected using an area detector with a 300 s exposure. The crystalline coherence length normal to the (hkl) planes, *D_hkl_*, was calculated using the Scherrer equation [25]:

(6) $$D_{hkl}=\frac{K\lambda }{\beta cos\theta }$$

Here, K = 0.89, β is the diffraction-peak full width at half maximum after correction for instrumental broadening, expressed in radians of 2θ, and θ is the Bragg angle. *D*_110_ was calculated from the α-iPP (110) peak. SAXS covered scattering vectors from 0.10 to 2.50 nm^−1^. After background subtraction and Lorentz correction, the long period, *L_p_*, was calculated from the principal peak position, *q*_max_, of I(q)q^2^ versus q. The full width at half maximum (FWHM) of that peak was extracted using identical peak-processing conditions [26]:

(7) $$L_{p}=\frac{2\pi }{q_{max}}$$

The apparent whole-sample PP crystallinity after the standardized DSC thermal cycle, *X_c_*, was converted to a mass fraction, *w_c_* = *X_c_*/100. Reference densities of *ρ_c_* = 0.936 g/cm^3^ and *ρ_a_* = 0.850 g/cm^3^ for PP crystalline and amorphous regions, respectively, were used to calculate the crystalline volume fraction, *φ_c_*, in a two-phase reference model [26,27]. Combining *φ_c_* with the SAXS long period, *L_p_*, of the compression-molded specimens gave *d_c_* = *φ_c_ L_p_* and *d_a_* = (1 − *φ_c_*)*L_p_*, combined quantities corresponding to the crystalline and amorphous reference fractions. These quantities jointly characterize the whole-sample crystalline fraction under the specified thermal history and the characteristic stacking scale. They are not the actual crystalline- and amorphous-layer thicknesses within local lamellar stacks.

(8) $$\varphi_{c}=\frac{\frac{w_{c}}{\rho_{c}}}{\frac{w_{c}}{\rho_{c}}+\frac{1-w_{c}}{\rho_{a}}}$$

(9) $$d_{c}=L_{p}\varphi_{c},d_{a}=L_{p}-d_{c}$$

### 2.6. Morphological and Optical Characterization

Etched HICP and RICP fracture surfaces were examined using a JSM-7500F field-emission scanning electron microscope (JEOL, Akishima, Japan) at an accelerating voltage of 1 kV. CICP was examined using a Nova NanoSEM 450 field-emission scanning electron microscope (FEI, Hillsboro, OR, USA) at 5 kV. At least five different regions were imaged for each sample. Crystalline organization in compression-molded films was recorded at room temperature using a BX51-P polarized optical microscope (Olympus, Tokyo, Japan). Haze was measured using a Haze-Gard series instrument (BYK-Gardner, Geretsried, Germany) [28]. Five films approximately 0.20 mm thick were selected for each material. Three positions per film were measured and averaged; each film was treated as the statistical unit for calculating the mean and sample standard deviation (*n* = 5).

### 2.7. Dynamic Mechanical Analysis and Rheology

DMA was performed in tension mode on a Q800 instrument (TA Instruments, New Castle, DE, USA) at 1 Hz, with a heating rate of 3 °C/min over −80–150 °C. Specimens measuring 30 mm × 4 mm were cut from 1.0 mm compression-molded sheets. Storage modulus, E′, and loss factor, tan δ, were recorded. Values of E′ at −50, 25, and 100 °C and characteristic relaxation temperatures in the tan δ spectra were extracted.

Dynamic rheology was measured on an MCR 302 rotational rheometer (Anton Paar, Graz, Austria) using 25 mm parallel plates with a 1.0 mm gap at 200 °C. The strain amplitude was 1%, and the angular-frequency range was 0.1–600 rad/s. Complex viscosity, η*, storage modulus, G′, and loss modulus, G″, were recorded; η* and G′ were extracted at 1 and 100 rad/s.

### 2.8. Electrical Properties and Charge Behavior

Dielectric responses were measured using a Concept 80 broadband dielectric spectrometer (Novocontrol, Hundsangen, Germany). Before testing, 20 mm-diameter gold electrodes were vapor-deposited on both film surfaces. Scans over 10^−1^–10^6^ Hz at 30, 70, and 90 °C recorded relative permittivity, ε′, and dielectric loss, tan δ. Values of ε′ at 1 kHz for each temperature and tan δ at 90 °C and 0.1 Hz were extracted.

High-field conduction was measured with reference to IEC 62631-3-1 using a three-electrode configuration and a 6517B electrometer (Keithley, Cleveland, OH, USA) [29]. Measurements were performed at 30, 70, and 90 °C. The electric field was increased stepwise from 5 to 50 kV/mm, and the current was recorded after a 10 min hold at each step. Current density, j, was calculated to obtain j–E curves. The characteristic transition field at each temperature and the current density at 30 °C and 50 kV/mm were extracted.

TSDC measurements were performed using a TSDC system (Novocontrol, Hundsangen, Germany). Specimens were heated to 140 °C and polarized under a 40 kV/mm DC field for 30 min. They were rapidly cooled to −80 °C while maintaining the field, short-circuited for 5 min, and heated to 150 °C at 3 °C/min while the depolarization current was recorded. The characteristic trap depth, *E_t_*, was calculated using a FWHM approximation for principal peaks with a complete FWHM. The depolarization current was integrated over the measurement temperature range to obtain the released charge. Normalization by the elementary charge, e, and effective specimen volume, Sd, gave the equivalent number density of thermally released carriers, *N_t_* [30]:

(10) $$E_{t}=\frac{2.47k_{B}{T_{p}}^{2}}{\Delta T}$$

(11) $$N_{t}=\frac{60\int I(T)dT}{\beta_{h}eSd}$$

Here, *k_B_* is the Boltzmann constant, *T_p_* is the absolute temperature of the principal peak, and ΔT is the temperature difference between its two half-height points, i.e., the FWHM. I(T) is the depolarization current, *β_h_* is the heating rate, e is the elementary charge, S is the effective electrode area, and d is the specimen thickness. I(T), S, and d are entered in A, m^2^, and m, respectively. The factor 60 converts the heating rate from K/min to K/s.

Net space-charge distributions through the specimen thickness were measured using a PEA system (FiveLab, Yokohama, Japan) [31]. Measurements were performed at room temperature. A 40 kV/mm DC field was applied for 2400 s, after which the voltage was removed and the specimen was short-circuited for 600 s. Space-charge distributions and their evolution were recorded continuously. The peak local charge density after 2400 s of polarization was extracted.

DC breakdown was measured using a laboratory-built high-voltage system with reference to IEC 60243-1 and IEC 60243-2 [32,33]. Specimens approximately 0.20 mm thick were immersed in silicone oil between 25 mm-diameter brass sphere–sphere electrodes. Voltage was increased continuously at 1 kV/s until breakdown. At least 10 valid results were obtained for each material and analyzed using a two-parameter Weibull distribution [34,35]:

(12) $$P(E)=1-exp[-{\left(\frac{E}{\alpha }\right)}^{\beta }]$$

Here, P(E) is the cumulative probability of breakdown failure, E is the breakdown field, α is the characteristic breakdown strength corresponding to a cumulative failure probability of 63.2%, and β is the shape parameter describing relative dispersion.

### 2.9. Mechanical Testing

Tensile and flexural tests were performed using an electronic universal testing machine (ZwickRoell, Ulm, Germany). Tensile properties were measured according to GB/T 1040.2-2022 [36], with stress and strain defined according to ISO 527-1:2012 [37]. For HICP and RICP, which exhibited yield peaks, tensile strength was represented by the first local stress maximum. For CICP, which lacked a distinct yield peak, the maximum tensile stress reached during displacement loading was reported [37]. Type 1A dumbbell specimens had a narrow-section width of approximately 10 mm, a thickness of approximately 4 mm, and an initial grip separation of 115 mm. Tensile modulus was measured at 1 mm/min, followed by continued loading at 50 mm/min. Nominal strain in the tensile curves was represented by the crosshead displacement increment divided by the initial grip separation.

Flexural properties were measured by three-point loading according to GB/T 9341-2008 [38]. Specimens measured 80 mm × 10 mm × 4 mm; the span was 64 mm, and the test speed was 2 mm/min. Flexural modulus and flexural stress at the specified deflection were obtained.

Notched Charpy impact properties were measured using a HIT25P impact tester (ZwickRoell, Ulm, Germany) according to GB/T 1043.1-2008 [39]. Type A notches, 2 mm deep, were machined in 80 mm × 10 mm × 4 mm specimens using an XQZ-1 notching machine (Chengde Jinjian Testing Instrument Co., Ltd., Chengde, China); the span was 62 mm. Tests were performed at 23, 0, and −20 °C, and failure types were recorded. Tensile and flexural tests each included five valid specimens per material. Impact tests included 10 specimens per material at each temperature. Results are reported as mean ± standard deviation.

### 2.10. Data Processing

Data processing and plotting were performed using Origin 2025b SR1 (build 10.250234; OriginLab Corporation, Northampton, MA, USA).

## 3. Results and Discussion

### 3.1. Molecular Sequences and Total Ethylene Content

Figure 2 shows representative normalized ^13^C NMR spectra of the three ICPs. Labels 1–13 and the accompanying list identify the principal resonance regions and sequence assignments [20]. The *S_αα_* signal at 47.5–44.5 ppm corresponds to methylene environments between adjacent propylene units. The *T_ββ_* signal at 28.9 ppm and *P_ββ_* signal at 21.8–21.0 ppm correspond to PPP sequences. *T_δδ_* at 33.2 ppm and *S_ββ_* at 24.9–24.0 ppm correspond to EPE and PEP sequences, respectively. *S_δδ_* at 29.9 ppm corresponds to methylene environments within consecutive ethylene sequences. The 30.8–30.9 ppm region contains overlapping *T_βδ_*/*S_γγ_* responses, including contributions from PPE/PEEP sequences [20,40]. Together, these resonances reflect the coexistence of consecutive propylene sequences and different ethylene-neighboring environments.

Integration of the resonance regions gave total ethylene contents of 11.4 ± 0.4, 13.7 ± 0.4, and 23.3 ± 0.2 wt% for HICP, RICP, and CICP, respectively (Table 2). The mean contents of HICP and RICP differed by 2.3 percentage points. Considering the polymerization route, ethylene introduced during the first stage for RICP forms part of the matrix chains and can alter the regularity and crystallizability of some propylene sequences [41]. For CICP, which has a higher total ethylene content, the coexistence of ethylene-rich components and crystallizable PP chain populations is particularly important. Its compositional basis requires further examination through molar-mass and crystallizability distributions.

### 3.2. Molar Mass and Crystallizability Distributions

All three materials exhibited broad molar-mass distributions (Figure 3 and Table 3). The ${\bar{M}}_{w}$ values of HICP, RICP, and CICP were 330.1 ± 10.0, 400.1 ± 15.2, and 311.0 ± 6.4 kg/mol, respectively, with corresponding *Đ_M_* values of 9.85 ± 0.45, 10.59 ± 0.43, and 8.41 ± 0.18. The ${\bar{M}}_{w}$ of RICP was 21.2% higher than that of HICP; the mean difference was 70.0 kg/mol, with a 95% confidence interval of 38.9–101.1 kg/mol (two-sided Welch’s *t*-test, *p* = 0.0044). Under the same catalyst system and principal polymerization conditions, ethylene copolymerization in the first stage was accompanied by an increased contribution from higher-molar-mass components, while the broad distribution was retained.

TREF and CFC fractionation were used to further distinguish the crystallizability of the different sequence structures. The TREF results showed an SF content of 18.56 ± 0.98 wt% and a main elution peak at 124.9 ± 0.9 °C for HICP. For RICP, the SF content increased to 27.26 ± 1.46 wt%, and the main peak shifted to 112.1 ± 1.4 °C (Figure 4 and Table 4). Compared with HICP, RICP had a 2.3-percentage-point increase in mean total ethylene content and an 8.70-percentage-point increase in SF content. These changes indicate that first-stage copolymerization modified both matrix sequence regularity and component crystallizability, increasing the population of chains in the low- and intermediate-crystallizability ranges. CICP had an SF content of 73.31 ± 0.60 wt% while retaining a high-temperature eluting component near 122.9 ± 0.3 °C, indicating the predominance of low-crystallizability or rubber-rich chains alongside a small population of highly regular PP chains. The shoulders and broadening on the low-temperature side of the main high-temperature peak reflect overlapping elution of chain populations with similar crystallizability but different sequence structures, associated with differences in comonomer distribution and isotactic propylene sequence length [7].

CFC further revealed the coupled distribution of elution temperature and molar mass (Figure 5). The main HICP signal was concentrated in the high-temperature elution region and was relatively separate from the low-temperature soluble fraction. For RICP, the distribution shifted toward lower temperatures, and the intermediate-component band at approximately 80–100 °C broadened, reflecting a crystallizability distribution extending from the highly crystalline matrix toward low-crystallizability components. Previous fractionation and recombination studies have shown that intermediate copolymer fractions can modify phase separation and interfacial morphology between rubber-rich and crystalline regions [7]. Thus, the increased population of intermediate-crystallizability chains in RICP provides a molecular basis for a more gradual compositional transition between PP-rich and rubber-rich regions. CICP was dominated by the low-temperature soluble fraction, with a weaker continuous distribution in the intermediate-temperature region and a retained high-crystallizability component at approximately 123 °C. Its composition was therefore dominated by low-crystallizability components coexisting with a small population of highly regular PP chains. The progression from relatively separate high- and low-crystallizability components in HICP, through increased intermediate components in RICP, to predominantly low-crystallizability components in CICP reveals the compositional basis for the differences in crystalline support and phase connectivity discussed below [8].

### 3.3. Crystalline Framework and Lamellar Organization

Differences in the distribution of crystallizable chains were further reflected in crystalline content and crystal thermal stability (Figure 6 and Table 5). HICP exhibited relatively narrow melting and crystallization peaks, with *T_m_*, *T_c_*, and apparent PP crystallinity *X_c_* of 166.5 ± 0.8 °C, 116.5 ± 0.1 °C, and (51.9 ± 0.9)%, respectively, corresponding to a relatively well-developed crystalline framework formed by the highly regular PP matrix. For RICP, *T_m_* and *T_c_* decreased to 146.8 ± 0.4 and 107.4 ± 0.5 °C, respectively; *X_c_* was (31.7 ± 0.7)%, and the melting peak broadened markedly. These changes are consistent with the lower-temperature TREF main peak and the increased intermediate-crystallizability fraction revealed by CFC. They indicate that ethylene copolymerization shortens consecutive isotactic propylene sequences, restricts the crystallization of regular chains and lamellar perfection, and broadens the distribution of crystal perfection [7,42].

CICP had an *X_c_* of only (13.3 ± 0.5)%, whereas its *T_m_* remained as high as 164.7 ± 0.2 °C. The combination of a low whole-sample melting enthalpy and a high melting peak temperature indicates a small PP crystalline fraction, while the remaining regular propylene sequences can still form crystals with relatively high thermal stability [8].

In the one-dimensional WAXS patterns, peaks 1, 3, 4, 5, 6, 7, and 8 correspond to the (110), (040), (130), (111), (−131)/(041), (060), and (220) reflections of α-iPP, respectively, whereas peak 2 corresponds to the β-iPP (300) shoulder (Figure 7a) [43,44,45]. The α form predominated in all three materials. A weak shoulder associated with β(300) was also discernible in RICP, indicating a polymorphic composition different from those of HICP and CICP. The *D*_110_ values of HICP, RICP, and CICP were 14.45 ± 0.67, 11.91 ± 0.54, and 12.38 ± 0.25 nm, respectively (Table 6). The smaller coherent crystalline dimension of RICP is consistent with the reduction in consecutive isotactic sequences following matrix randomization. Although the crystalline diffraction of CICP was weak relative to its diffuse background, *D*_110_ remained approximately 12 nm, consistent with a low crystalline fraction whose residual crystalline regions retain appreciable internal order.

All two-dimensional WAXS patterns showed diffraction rings with relatively uniform azimuthal intensity, indicating approximately random orientation in the measurement plane (Figure 8). The diffraction rings of RICP were comparatively diffuse. Distinct α-form diffraction rings remained discernible in CICP, indicating that its residual PP crystalline regions retained identifiable lattice order despite the low whole-sample crystalline fraction.

Lorentz-corrected SAXS curves were used to further characterize the long-period structure of lamellar stacks. The representative main peaks of the three samples were located at *q*_max_ ≈ 0.40, 0.43, and 0.44 nm^−1^, corresponding to *L_p_* values of 15.71, 14.61, and 14.28 nm, respectively. The mean *L_p_* values from three measurements were 15.54 ± 0.43, 14.73 ± 0.28, and 14.29 ± 0.06 nm, respectively (Figure 7b and Table 6). The combined quantity *d_c_* decreased from 7.69 ± 0.30 nm for HICP to 4.36 ± 0.17 nm for RICP, a reduction of 43.3%, whereas *d_a_* increased from 7.85 ± 0.21 to 10.37 ± 0.16 nm. These changes reflect both a reduced crystalline reference fraction and a smaller *L_p_*. The full width at half maximum (FWHM) of the main SAXS peak increased from 0.227 ± 0.003 to 0.310 ± 0.009 nm^−1^. The shift in the correlation peak toward higher q and its broadening indicate a smaller characteristic stacking distance accompanied by changes in the distribution of stacking scales and their order [46]. Together with the broader DSC melting peak and smaller WAXS coherent dimension, these results reveal the effect of matrix randomization on the crystalline framework.

For CICP, the combined quantities *d_c_* and *d_a_* were 1.75 ± 0.06 and 12.54 ± 0.11 nm, respectively. Calculated jointly from the whole-sample long period and volume crystallinity, *d_c_* represents the mean effective contribution of crystalline layers in the multiphase long-period model. Because the crystallinity of CICP was only 13.3%, this parameter primarily reflects the sparse crystalline framework within the overall structure. Together, *D*_110_ and *T_m_* indicate that CICP contains a small PP crystalline fraction whose residual crystalline regions retain appreciable internal order and thermal stability, providing dispersed crystalline support for the large proportion of low-crystallizability components.

POM revealed corresponding differences in crystalline organization at the micrometer scale (Figure 9). HICP exhibited strong birefringent textures and well-developed spherulitic organization. RICP showed finer birefringent units, smaller bright regions, and overlapping boundaries, consistent with its lower *T_c_*, smaller *D*_110_, and broader SAXS stacking correlation peak. The CICP field of view was generally dark, with only dispersed, weak birefringent textures, corresponding to a small population of PP crystalline regions dispersed within abundant low-crystallizability components. Thermal analysis, X-ray scattering, and POM show that the differences among the three materials extend from the fraction of crystallizable chains to internal crystalline order, lamellar stacking, and micrometer-scale crystalline organization: HICP forms a relatively well-developed crystalline framework; RICP has reduced crystalline content and finer organization; and CICP retains dispersed PP crystalline supports. This spatial partitioning of crystalline and amorphous components further influences connectivity between PP-rich and rubber-rich regions.

### 3.4. Multiphase Morphology and Optical Response

Selectively etched fracture surfaces revealed different patterns of phase connectivity among the three materials (Figure 10). In HICP, a relatively continuous, dense matrix surrounded comparatively isolated, rounded domains with clear boundaries, producing a discrete sea–island morphology. Together with the fractionation and crystallization results, this corresponds to discrete rubber-rich domains embedded in a highly regular PP matrix. Discrete domains remained identifiable in RICP, but more sheet-like, fibrillar, and wrinkled structures appeared at domain edges and between matrix regions, giving the interfacial transition a more irregular morphology. This change corresponds to the broadened intermediate-component band at approximately 80–100 °C in CFC, the reduced PP crystalline fraction, and the finer crystalline framework. Intermediate-crystallizability chains provide molecular conditions for connecting compositionally different regions, while the reduced crystalline fraction and finer crystalline regions increase the space occupied by amorphous chains. Together with the connected morphology observed on the fracture surfaces, these features support enhanced physical connectivity and a greater tendency toward interfacial bridging between PP-rich and rubber-rich regions [7,8].

After mild etching, CICP exhibited labyrinthine interconnected pores and a continuous residual framework throughout the field of view (Figure 10), in marked contrast to the predominantly isolated domains in HICP. Its high SF content, low PP crystalline fraction, and interconnected morphology jointly indicate high spatial continuity of the low-crystallizability/rubber-rich components, with a small population of PP crystalline regions dispersed within them.

Haze describes the fraction of total transmitted light that is scattered at wide angles and depends jointly on refractive-index fluctuations, the size and number density of scattering structures, and film thickness [47,48]. The mean film thicknesses of HICP, RICP, and CICP were 0.214, 0.221, and 0.201 mm, respectively, and their haze values were 97.7 ± 0.8%, 77.7 ± 0.7%, and 64.6 ± 0.7%, respectively (Table 7). The well-developed crystalline organization and discrete domains in HICP generate substantial optical heterogeneity and thus strong wide-angle scattering. Crystal refinement and a broader interphase transition in RICP reduce the contribution of large-scale scattering structures, consistent with the reduced haze associated with finer crystalline organization and improved domain dispersion in multiphase polypropylene systems [47,48]. The lowest haze of CICP corresponds to its low crystalline fraction and dispersed crystalline regions. Thus, the macroscopic optical response supports the changes in structural scale and phase organization observed by POM and SEM.

### 3.5. Dynamic Load Bearing and Segmental Relaxation

DMA reflects the combined constraints imposed by the crystalline framework and multiphase organization on solid-state segmental motion and dynamic load bearing (Figure 11 and Table 8). The mean E′ values of HICP at −50, 25, and 100 °C were 4490 ± 190, 1738 ± 66, and 368 ± 21 MPa, respectively, all higher than those of RICP and CICP. At 25 °C, E′ decreased to 970 ± 18 MPa for RICP and 74.6 ± 0.4 MPa for CICP; at 100 °C, these values were 190 ± 10 and 5.5 ± 0.1 MPa, respectively. The reduced crystalline fraction and finer crystalline regions of RICP weaken its solid-state rigidity. Above room temperature, deformation of CICP is dominated by its large rubber-rich fraction, with dispersed PP crystalline regions providing limited physical support [49].

In the representative tan δ spectrum of HICP, relaxations of the rubber-rich phase and the amorphous regions of the PP matrix appeared near −31.2 and 18.3 °C, respectively, as relatively separate responses. The corresponding peak temperatures of RICP were −38.0 and 17.9 °C, with increased relative damping, consistent with more amorphous chains and weaker crystalline constraints. CICP exhibited a principal peak near −8.4 °C in its overlapping low-temperature relaxation region and retained a shoulder at approximately −36.2 °C, reflecting overlapping relaxations of amorphous chains with different compositions. In heterophasic ethylene–propylene copolymers, finer phase dispersion and stronger interphase coupling can be accompanied by merging low-temperature relaxation peaks [8,49]. Considered together with the SEM results, the broadened response of CICP reflects soft-phase-dominated segmental motion and stronger relative damping.

In the high-temperature region, the α* relaxation peaks in the representative HICP and RICP curves were located at 111.4 and 96.0 °C, respectively, corresponding to thermally activated motion within the PP crystalline structure [50]. The lower peak temperature of RICP is consistent with its finer crystalline framework and weaker constraints. With increasing temperature, the storage modulus of CICP continued to decrease while its loss factor increased, reflecting the combined effects of weakening crystalline support and enhanced segmental motion.

### 3.6. Melt Viscoelastic Response and Interphase Constraints

After the crystals melt, relaxation of molecular chains and phase domains becomes the principal source of the viscoelastic response. At 200 °C, above the PP melting peak temperatures of all three materials, η* decreased with increasing angular frequency, exhibiting dynamic shear thinning. Both η* and G′ followed the order CICP > RICP > HICP (Figure 12 and Table 9). At 1 rad/s, the η* values of HICP, RICP, and CICP were 9.0 ± 0.3, 16.3 ± 1.2, and 21.4 ± 0.9 kPa·s, respectively, and their G′ values were 4.2 ± 0.2, 8.0 ± 0.7, and 10.3 ± 0.4 kPa, respectively. Increasing the angular frequency to 100 rad/s decreased η* and increased G′ without changing the order among the materials.

The low-frequency response reflects chain and domain relaxation over longer timescales. Similar PP/EPR/PE reactor alloys can exhibit both relatively fast PP-chain relaxation and slower relaxation of rubber-rich domains and interfaces above the PP melting point [51]. The higher ${\bar{M}}_{w}$ of RICP helps delay terminal chain relaxation. Together with the increased intermediate-crystallizability chain population revealed by CFC and the extended interphase connectivity observed by SEM, its higher low-frequency η* and G′ also reflect stronger long-timescale constraints between components. CICP had the lowest mean ${\bar{M}}_{w}$ but the highest low-frequency η* and G′, further indicating the important contributions of component composition and spatial organization to the melt response. Its high rubber-rich fraction and strong phase continuity, together with the component viscosity ratio, interphase coupling, and slow interfacial relaxation, maintained high melt viscosity and elasticity [52,53].

Solid-state DMA E′ followed the order HICP > RICP > CICP, opposite to the order of low-frequency melt G′, indicating that different structural factors dominate the viscoelastic response in the two states. The solid-state response is governed mainly by the crystalline framework and constraints on amorphous chains, whereas the melt response is determined jointly by molecular-chain and domain relaxation. Reduced solid-state rigidity can therefore coexist with a strong long-timescale melt viscoelastic response.

### 3.7. Dielectric Response and High-Field Charge Transport

In addition to affecting load transfer and segmental relaxation, the multiphase organization described above can influence insulation behavior by modifying local polarization and the conditions for carrier migration. Representative broadband dielectric spectra of the three ICPs are shown in Figure 13. Over 10^−1^–10^6^ Hz, ε′ ranged from 2.01 to 2.15, consistent with the low permittivity and weak frequency dispersion characteristic of polyolefins. At 30 °C and 1 kHz, the ε′ values of HICP, RICP, and CICP were 2.138 ± 0.006, 2.085 ± 0.012, and 2.096 ± 0.003, respectively. All decreased upon heating to 70 and 90 °C and followed the order HICP > RICP > CICP (Table 10). Thermal expansion reduces the number density of polarizable groups and is an important reason for the decrease in ε′ with increasing temperature [54]. Differences among the materials also relate to phase composition and packing: the high crystalline content of HICP helps maintain a relatively dense PP matrix, whereas the increased proportion of low-crystallizability chains and the dispersed crystalline framework in RICP and CICP alter the volume distribution of polarizable groups [55].

At 30 and 70 °C, tan δ was on the order of 10^−5^–10^−3^ across most of the frequency range, with localized increases at low frequencies. At 90 °C, the low-frequency loss of HICP increased markedly, reaching (5.1 ± 0.4) × 10^−3^ at 0.1 Hz, compared with (1.2 ± 0.1) × 10^−3^ for RICP and (1.7 ± 0.1) × 10^−3^ for CICP. Low-frequency loss in polyolefins is sensitive to thermally activated carrier migration and interfacial charge accumulation [56,57]. The weaker high-temperature, low-frequency losses of RICP and CICP are consistent with their lower high-field current densities discussed below, indicating that charge migration and accumulation in these materials are jointly regulated by phase composition and local trapping environments.

Figure 14 shows representative j–E curves at different temperatures. On logarithmic axes, all curves exhibited a smaller slope at low fields and an increased slope at high fields, associated with field-dependent changes in electrode injection, trap occupancy, and migration across microregions [58,59]. Across all temperatures and fields tested, current densities in the representative curves followed the order HICP > RICP > CICP. As the temperature increased from 30 to 90 °C, the characteristic transition fields of HICP, RICP, and CICP decreased from 14.4 ± 0.6, 25.4 ± 1.5, and 33.9 ± 2.0 kV/mm to 9.5 ± 0.2, 16.1 ± 1.1, and 19.1 ± 0.7 kV/mm, respectively (Table 11). Heating enhances thermally activated carrier detrapping, shifting the nonlinear response to lower fields; the transition fields of RICP and CICP remained higher than those of HICP.

At 30 °C and 50 kV/mm, the current densities of HICP, RICP, and CICP were (1.4 ± 0.2) × 10^−7^, (5.3 ± 0.5) × 10^−8^, and (8.7 ± 0.9) × 10^−9^ A/m^2^, respectively. The latter two were approximately 37.6% and 6.2% of that of HICP. The discrete phase interfaces and crystalline–amorphous boundaries of HICP provide structural sites for local transport differences and charge accumulation [9]. The increased intermediate-crystallizability chain population and interphase transition regions in RICP modify the barriers and trapping environments encountered by carriers migrating across microregions. In CICP, dispersed PP crystalline regions and the constrained chains surrounding them connect with the continuous low-crystallinity/rubber-rich phase. Local differences in composition and chain packing can modify the conditions for carrier trapping and interphase migration [60]. Meanwhile, fewer abrupt, isolated interfaces also reduce local field concentration and interfacial injection.

To distinguish the trapping and detrapping processes involved in these current differences, the TSDC responses of the different phase organizations were examined (Figure 15). HICP exhibited a narrow, intense main peak. The main RICP peak shifted to a higher temperature and had a lower peak current, whereas CICP exhibited a broad high-temperature peak with a weaker low-temperature release peak. The main peak temperatures of the three materials were 92.0 ± 1.4, 105.3 ± 2.0, and 135.8 ± 1.1 °C, respectively. The characteristic trap depths of HICP and RICP were 0.92 ± 0.06 and 1.05 ± 0.03 eV, respectively (Table 12). The equivalent number density of thermally released carriers followed the order HICP > CICP > RICP, reflecting differences in the effective thermally released charge per unit volume under the specified polarization procedure and within the tested temperature range [61]. Peak-shape analysis of HICP and RICP indicates a higher characteristic detrapping barrier in the latter. The broad high-temperature response of CICP corresponds to a wider thermal-release temperature range and can be associated with the superposition of different local trapping environments and high-temperature segmental relaxation processes [57,62].

These changes correspond to the crystalline and interphase structures. The highly regular matrix and discrete phase interfaces of HICP correspond to a relatively concentrated thermal-release response. The finer crystals and increased population of intermediate-crystallizability chains in RICP diversify segmental constraints in crystalline–amorphous and interphase transition regions and are associated with the increase in characteristic trap depth. In CICP, dispersed PP crystalline regions and their surrounding constrained chains within the continuous low-crystallinity phase can create different local trapping environments [60]. During heating, the combined effects of charge detrapping and local segmental motion may spread the current response over a broad temperature range.

Under the thermally activated detrapping approximation, the characteristic residence time of a carrier in a trap can be expressed as [12,61]

(13) $$\tau_{t}=\tau_{0}exp(\frac{E_{t}}{k_{B}T})$$

where *τ*_0_ is the attempt time, and T is the absolute temperature. At 303 K and the same attempt frequency, calculations using the arithmetic means of the three *E_t_* measurements give a characteristic residence time for RICP approximately 1.5 × 10^2^ times that for HICP, illustrating the exponential effect of the detrapping barrier on charge residence. Overall carrier release and transport also depend on trap density, the local energy-level environment, and segmental motion. The high-field drift current density can be expressed as [59]

(14) $$j=qn_{f}\mu E$$

where q is the carrier charge; *n_f_* is the free-carrier concentration; and μ is the free-carrier mobility. Deeper traps can extend the residence time of captured carriers and reduce the fraction participating in long-range drift. Charge trapping near electrodes can also modify the local field and regulate subsequent injection. The combined effects of bulk trapping–detrapping and charge regulation near electrodes can explain the lower high-field currents of RICP and CICP [12].

### 3.8. Space-Charge Evolution and DC Breakdown

Local trapping and migration jointly affect the spatial distribution of net charge. The PEA results further reveal charge accumulation and dissipation along the thickness direction. After polarization at 40 kV/mm for 2400 s, HICP developed a pronounced charge-density gradient through its thickness and retained substantial residual charge after short-circuiting. Charge accumulation was weaker in RICP, while CICP maintained low charge levels during both polarization and short-circuiting (Figure 16). The local peak charge densities of HICP, RICP, and CICP were 71.8 ± 8.7, 28.7 ± 0.7, and 12.6 ± 0.3 C/m^3^, respectively (Table 12). Based on the group means, those of RICP and CICP were 60.0% and 82.5% lower than that of HICP, respectively.

TSDC characterizes thermal release under a specified thermoelectrical procedure, whereas PEA reflects the net-charge distribution along the thickness direction. Together with the j–E curves, they describe the relationships among charge trapping, migration, and accumulation [63]. The shallower traps in HICP provide weaker long-term carrier confinement, allowing charge to spread more readily into the bulk. Interfacial transitions and deeper traps in RICP favor restricted long-range migration. The broad high-temperature thermal-release response of CICP corresponds to its low high-field current and low bulk charge, indicating that local confinement and constraints on interphase migration jointly restrict effective long-range carrier transport.

Under a one-dimensional, macroscopically homogeneous-medium approximation, space charge and the local field obey the Poisson relation [64]:

(15) $$\frac{dE_{sc}(x)}{dx}=\frac{\rho_{sc}(x)}{\varepsilon }$$

(16) $$E_{loc}(x)=E_{0}+E_{sc}(x)$$

where *ρ*_sc_(x) is the space-charge density, ε = *ε*_0_*ε_r_* is the absolute permittivity, *E*_0_ is the applied mean field, and *E*_sc_(x) is the additional field generated by space charge. Space-charge density determines the rate at which the local electric field varies through the thickness. At a given applied voltage, the sign, spatial extent, and position of the charge jointly determine the magnitude of the additional field. By modifying the local field distribution, bulk space charge causes the peak field experienced within the material to deviate from the applied mean field. Thus, the lower bulk space charge in RICP and CICP provides a direct charge-behavior basis for reducing local field distortion and delaying high-field failure.

The Weibull distributions of DC breakdown strength are shown in Figure 17, and the corresponding parameters are listed in Table 13. The characteristic breakdown strengths α of HICP, RICP, and CICP were 226.2, 310.8, and 384.4 kV/mm, respectively, representing increases of 37.4% and 69.9% for RICP and CICP relative to HICP. Although the total ethylene contents of HICP and RICP differed by only 2.3 percentage points, RICP combined reduced rigidity with a substantial increase in breakdown strength, indicating that the crystalline and interfacial structural changes induced by matrix randomization favor improved high-field stability.

The β values of HICP, RICP, and CICP were 14.8, 5.6, and 10.5, respectively, and did not vary monotonically with characteristic breakdown strength. The higher β of HICP indicates a narrower spread of breakdown results, although its characteristic breakdown strength was the lowest. The lower β of RICP indicates greater relative dispersion in its breakdown results, potentially associated with heterogeneous local failure conditions in its random-copolymer matrix and multiphase transition structure. CICP combined the highest characteristic breakdown strength with lower relative dispersion than RICP.

Together, Table 12 and Table 13 show that both the increased characteristic trap depth of RICP and the broad high-temperature thermal-release response of CICP correspond to lower bulk space charge and higher characteristic breakdown strength. The equivalent number density of thermally released carriers did not follow the same ordering. The effect of traps depends on their energy levels and local structural environments; deeper trapping states restrict long-range carrier transport by extending local residence times [5,12]. Reorganization of the crystalline framework and interphase transition regions further regulates charge accumulation and the resulting additional field, enabling RICP and CICP to withstand higher applied electric fields despite their lower rigidity.

### 3.9. Mechanical Properties

Regulation of multiphase topology is ultimately reflected in the coupled responses of flexibility and high-field insulation stability. The macroscopic mechanical properties of the three materials are listed in Table 14. The tensile elastic modulus and flexural modulus of RICP were 38.8% and 38.7% lower than those of HICP, respectively, consistent with the reductions in PP crystalline fraction and DMA storage modulus. The corresponding moduli of CICP were 89.4 ± 4.9 and 72.8 ± 3.6 MPa, reflecting the dominant role of the continuous low-crystallinity/rubber-rich phase in macroscopic deformation, with dispersed PP crystalline regions providing physical support.

Figure 18 shows the tensile and flexural curves of the three materials. Both HICP and RICP exhibited a stress decrease after yielding, followed by an extended large-deformation stage and subsequent strain hardening, during which the stress increased again. Sustained post-yield deformation reflects the development of plastic deformation, while the later stress increase is associated with chain orientation and enhanced load bearing by the molecular network [44]. CICP showed no pronounced yield peak and continued to deform at low stress, reflecting deformation accommodation by its continuous flexible phase. In the flexural curves, HICP and RICP maintained higher initial slopes and stress levels, whereas CICP had a markedly lower initial slope, consistent with the flexural modulus differences in Table 14.

At 23 °C, all three materials exhibited partial break, with RICP showing the highest notched impact strength. At 0 and −20 °C, HICP and RICP changed to complete break, whereas CICP retained partial break and had a notched impact strength of 97 ± 4 kJ/m^2^ at −20 °C. Within the ductile regime, energy absorption in polypropylene/elastomer systems depends jointly on yield stress, the extent of plastic deformation, and crack propagation. Absorbed energy can increase with decreasing temperature while the material remains within this ductile deformation regime [65,66]. The low modulus of CICP and its retained partial-break behavior at −20 °C reflect the role of its continuous low-crystallinity/rubber-rich phase in accommodating deformation and regulating crack propagation.

### 3.10. Coordinated Mechanical–Electrical Performance and Multiscale Mechanisms

Figure 19 relates changes in molecular and crystalline structure to phase organization and macroscopic responses. The highly regular PP matrix of HICP forms continuous crystalline support, while the discrete rubber-rich phase primarily accommodates local deformation. The material therefore retains relatively high rigidity. Matrix randomization in RICP reduces the regularity of crystallizable sequences, while intermediate-crystallizability chains produce a more continuous compositional transition between PP-rich and rubber-rich regions [7,8]. Weaker crystalline support and enhanced interphase connectivity jointly modify load transfer and segmental constraints: the former reduces the solid-state modulus, while the latter corresponds to slower melt relaxation. This structural reorganization also modifies the local environments encountered by carriers crossing crystalline–amorphous boundaries and interphase transition regions and is associated with the lower high-field current and bulk charge of RICP.

Previous studies of reactor-made ICPs have shown that further increasing the rubber-phase fraction can change an improvement in AC breakdown performance into a marked deterioration [6]. In the present study, CICP retained a characteristic DC breakdown strength of 384.4 kV/mm at a total ethylene content of 23.3 wt%. Its continuous low-crystallinity/rubber-rich phase provides space for large deformation, while dispersed PP crystalline regions and their surrounding constrained chains form local supports. Constraints on chains adjacent to crystalline regions, together with differences in composition and packing between PP-rich and ethylene-rich regions, can modify local trapping environments; phase connectivity influences carrier migration between compositionally different regions [9,60]. Thermal release in CICP occurred mainly at higher temperatures. Together with the coexistence of dispersed crystalline regions and a continuous flexible phase, this supports a mechanism in which local trapping and constraints on interphase migration jointly restrict effective long-range carrier transport. Reduced net-charge accumulation in the bulk helps mitigate space-charge-induced local field distortion, allowing the flexible-phase-dominated material to retain high DC breakdown performance.

## 4. Conclusions

HICP and RICP were investigated alongside CICP as a high-phase-continuity reference. Examination of the successive relationships among molecular sequences, the crystalline framework, multiphase organization, charge transport, and macroscopic mechanical–electrical properties led to the following conclusions.

(1) Changes in matrix sequences are first manifested in the crystallizability distribution and crystalline support. Following the introduction of ethylene comonomer in the first polymerization stage, the main elution peak shifted from 124.9 °C in HICP to 112.1 °C in RICP, and the apparent PP crystallinity decreased from 51.9% to 31.7%, accompanied by a smaller WAXS coherent dimension and a broader SAXS stacking correlation peak. CICP was rich in the low-temperature soluble fraction and had an apparent PP crystallinity of 13.3% on a whole-sample mass basis. Its residual PP crystals retained a high melting temperature, combining a low crystalline fraction with high crystal thermal stability.

(2) The crystalline framework and chains of different crystallizabilities jointly regulate phase connectivity. HICP exhibited a highly regular PP matrix and discrete rubber-rich domains with relatively clear interfaces. Crystal refinement and intermediate-crystallizability components in RICP expanded the interphase transition region and produced interfacial bridging features. CICP combined a continuous low-crystallinity/rubber-rich phase with dispersed PP crystalline regions. Microscopy, DMA, and dynamic rheology jointly support these structural differences: weaker solid-state crystalline support reduces rigidity, while interphase chain coupling and continuity-related constraints can maintain a strong long-timescale melt viscoelastic response.

(3) Phase connectivity and local segmental confinement further affect charge trapping, migration, and accumulation. The characteristic trap depths of HICP and RICP were 0.92 and 1.05 eV, respectively, whereas CICP exhibited a broad high-temperature thermal-release response. After polarization at 40 kV/mm for 2400 s at room temperature, the local peak net-charge densities in the bulk were 71.8, 28.7, and 12.6 C/m^3^, respectively. Deeper traps extend carrier residence times, while fewer abrupt, isolated interfaces help reduce local field concentration. Together, these effects reduce bulk charge and are associated with the lower high-field currents of RICP and CICP. Weaker net-charge accumulation in the bulk helps reduce the additional field generated by space charge, providing a charge-behavior basis for delaying local high-field failure.

(4) These multiscale structural changes are ultimately reflected in simultaneous improvements in flexibility and DC insulation performance. Relative to HICP, RICP exhibited a 38.8% lower tensile elastic modulus and a 37.4% higher Weibull characteristic breakdown strength. CICP retained a characteristic breakdown strength of 384.4 kV/mm despite a total ethylene content of 23.3 wt%, an apparent PP crystallinity of 13.3%, and a tensile elastic modulus of only 89.4 MPa. Its continuous flexible phase accommodates large deformation, while dispersed PP crystalline regions provide physical support. Constrained chains surrounding crystalline regions and compositional transition environments can restrict long-range carrier transport through local trapping and constraints on interphase migration. This structure underpins the combination of high flexibility and high-field insulation performance in CICP despite its high ethylene content and low crystallinity.

## Figures and Tables

**Figure 1:**
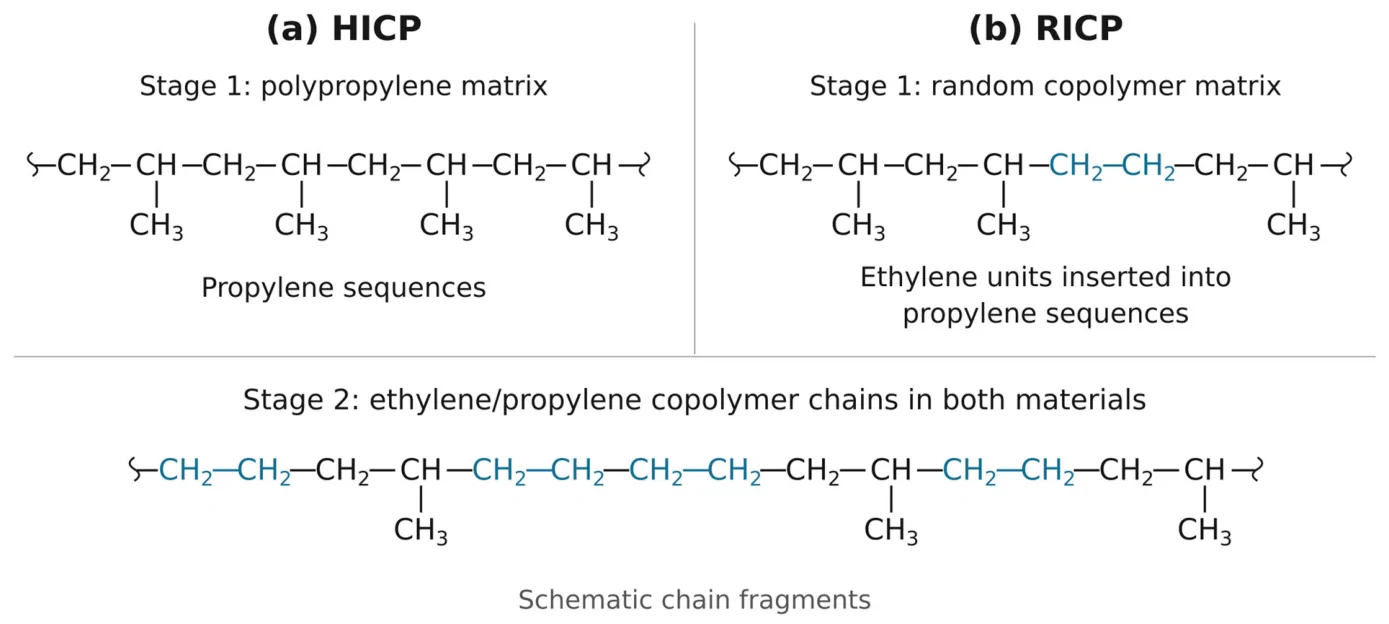
Schematic of the components and chain-segment structures produced by two-stage polymerization of (**a**) HICP and (**b**) RICP. The first stage forms a homopolypropylene matrix in HICP and a random-copolymer matrix containing ethylene comonomer units in RICP. Both materials form ethylene–propylene copolymer segments in the second stage. Blue indicates ethylene-derived –CH_2_–CH_2_– units; propylene-derived units are –CH_2_–CH(CH_3_)–. Wavy bonds indicate chain continuation. The illustrated segments do not represent the actual unit proportions.

**Figure 2:**
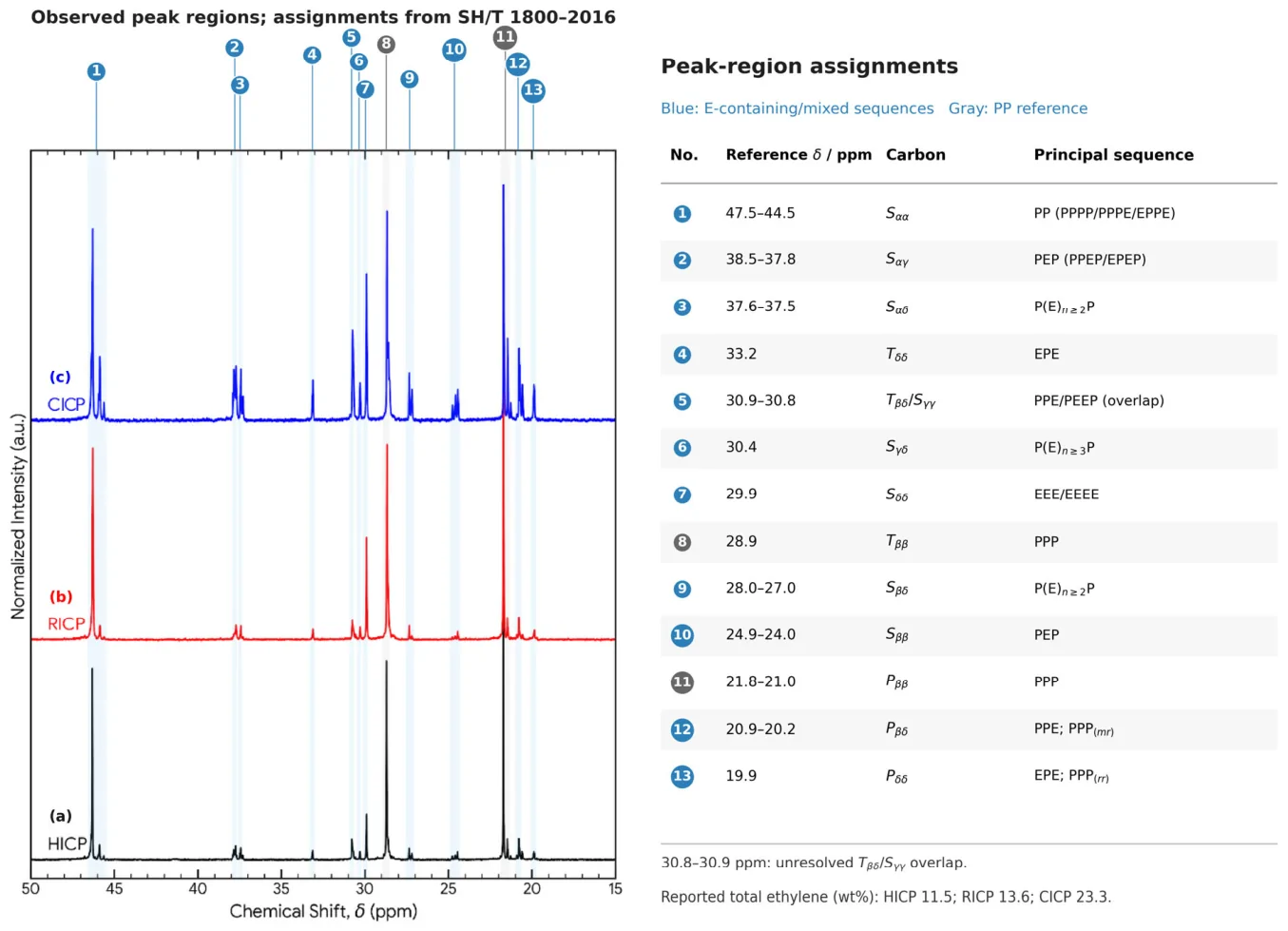
Representative normalized stacked ^13^C NMR spectra and principal resonance-region assignments for (**a**) HICP, (**b**) RICP, and (**c**) CICP. Labels 1–13 correspond to the peak-assignment list on the right [20]. The total ethylene contents shown in the figure correspond to the measurements marked a in Table 2.

**Figure 3:**
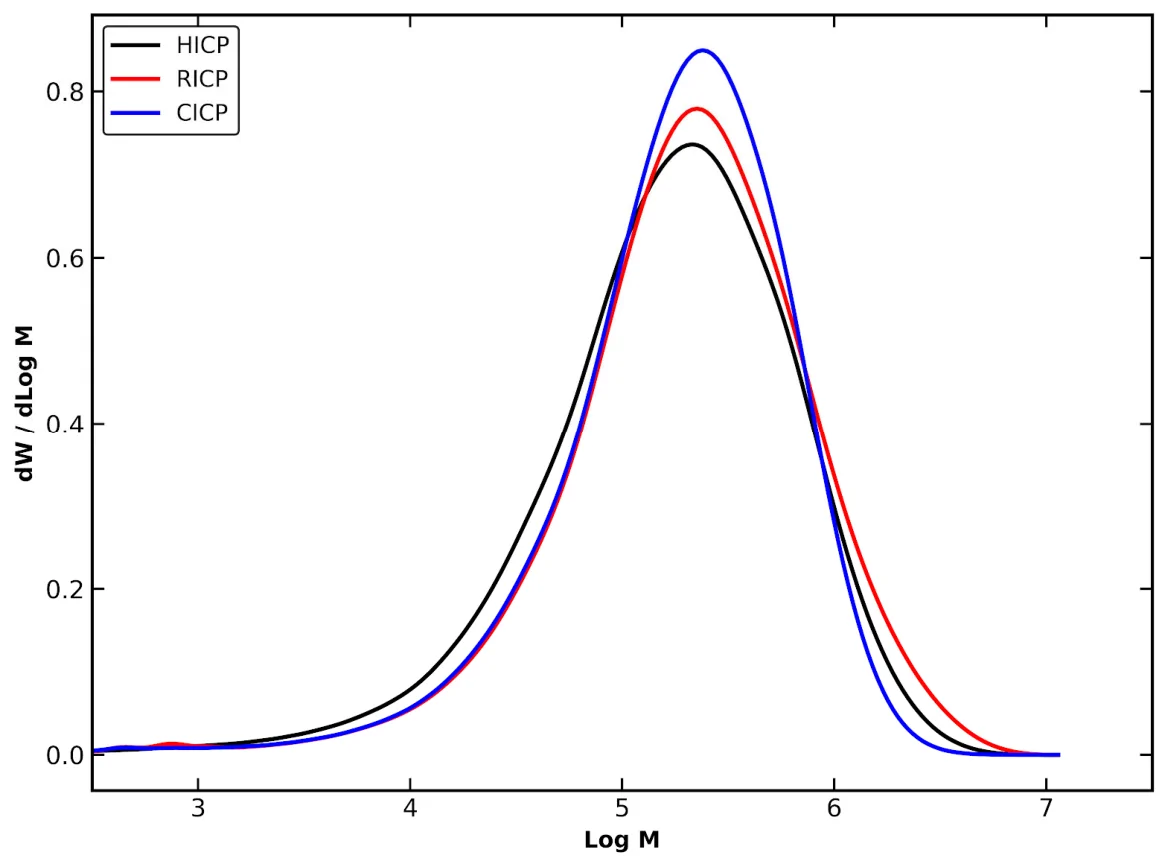
Representative molar-mass distribution curves of HICP, RICP, and CICP. The horizontal axis is the base-10 logarithm of the molar mass M expressed in g/mol, and the vertical axis is the distribution density dW/dlog M of the mass fraction W with respect to log M.

**Figure 4:**
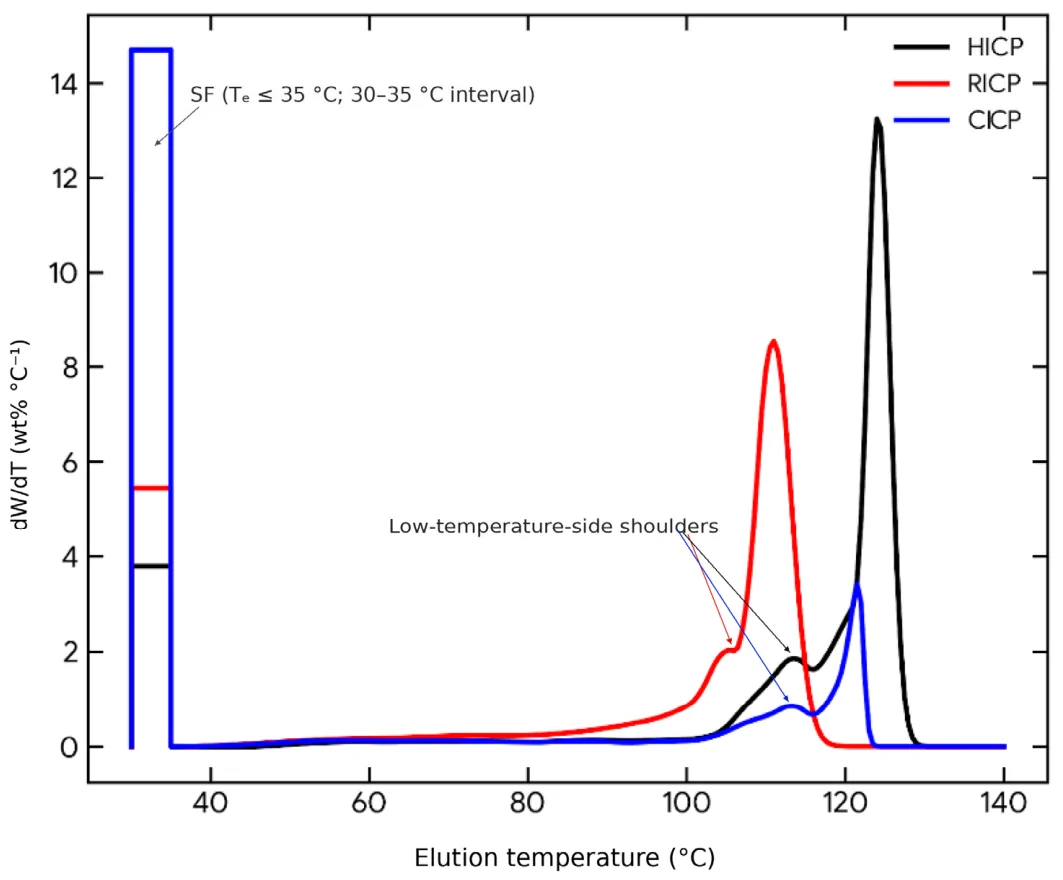
Representative TREF elution curves of HICP, RICP, and CICP. The rectangle at 30–35 °C is an area-equivalent representation of the SF: its height is the SF mass fraction divided by 5 °C, so its integrated area equals the corresponding SF mass fraction. Arrows indicate shoulders on the low-temperature side of the main peak.

**Figure 5:**
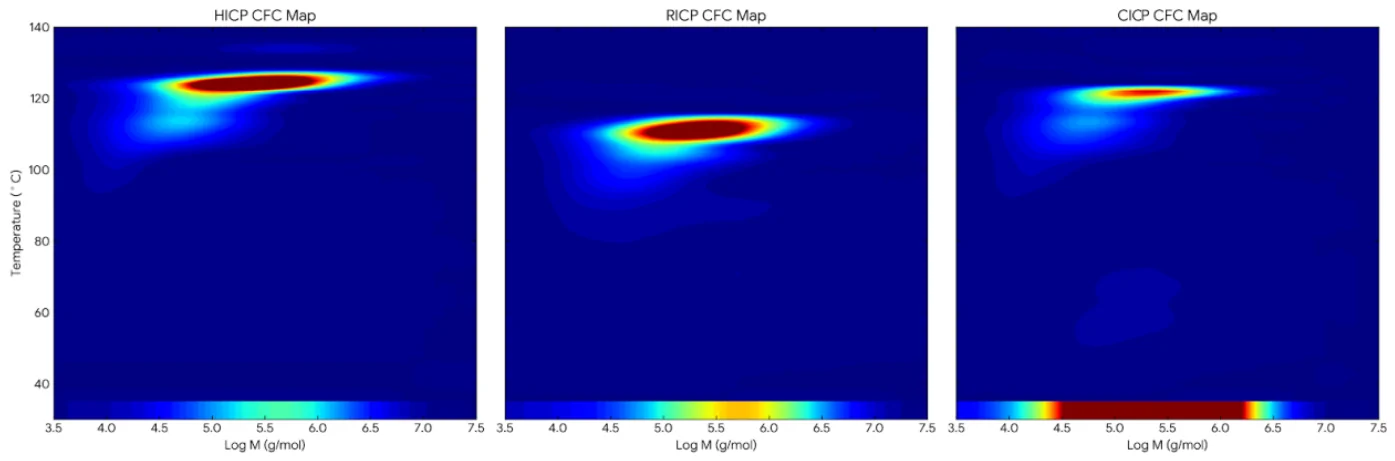
Representative CFC contour maps of elution temperature versus molar mass for HICP, RICP, and CICP.

**Figure 6:**
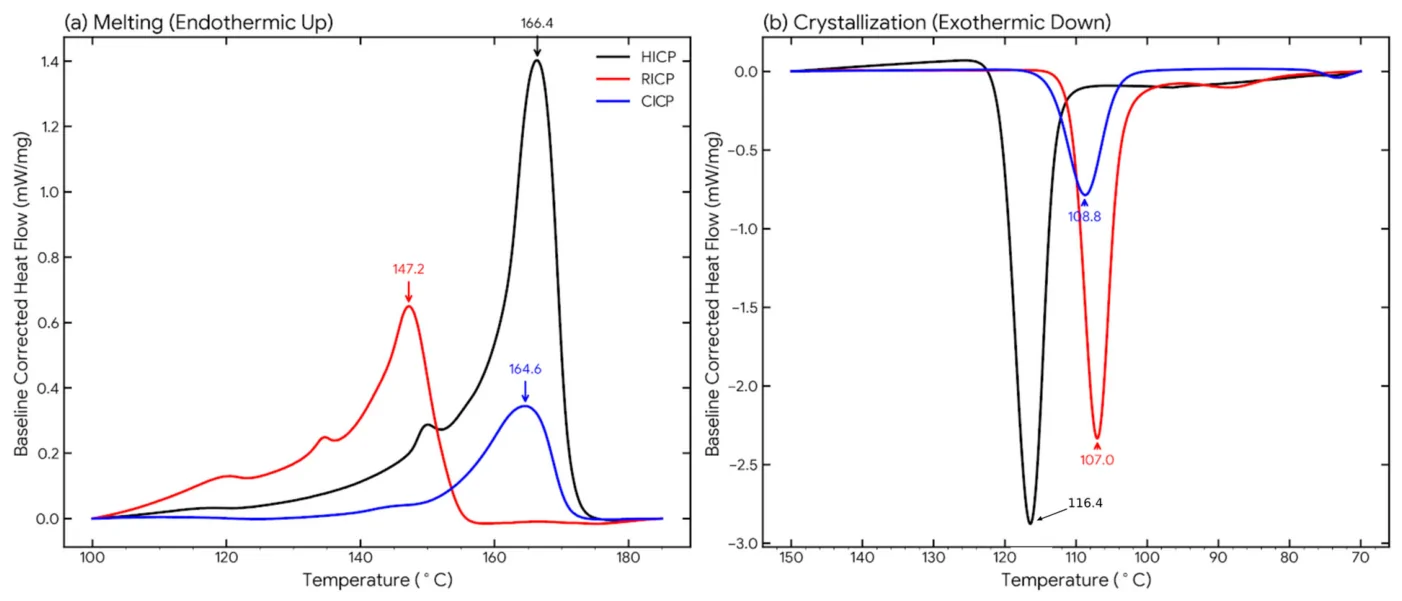
Representative DSC curves of HICP, RICP, and CICP: (**a**) melting during the second heating scan and (**b**) crystallization during cooling.

**Figure 7:**
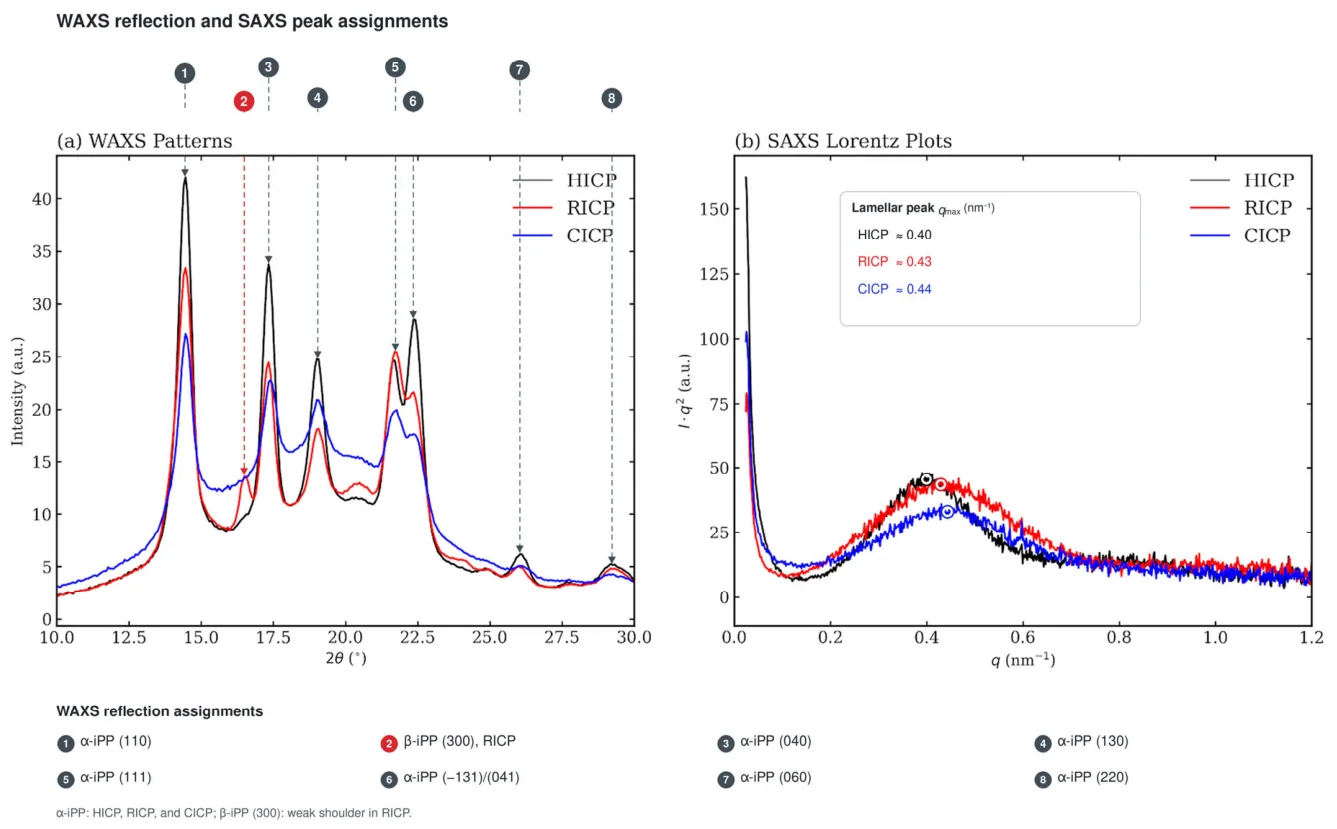
Representative (**a**) WAXS patterns and (**b**) Lorentz-corrected SAXS curves of HICP, RICP, and CICP. The assignments of peaks 1–8 in (**a**) are given in the text. In (**a**), gray numbered circles identify α-iPP reflection assignments, and the red circle identifies the β-iPP (300) shoulder in RICP. Open circles in (**b**) mark the principal SAXS peak positions.

**Figure 8:**
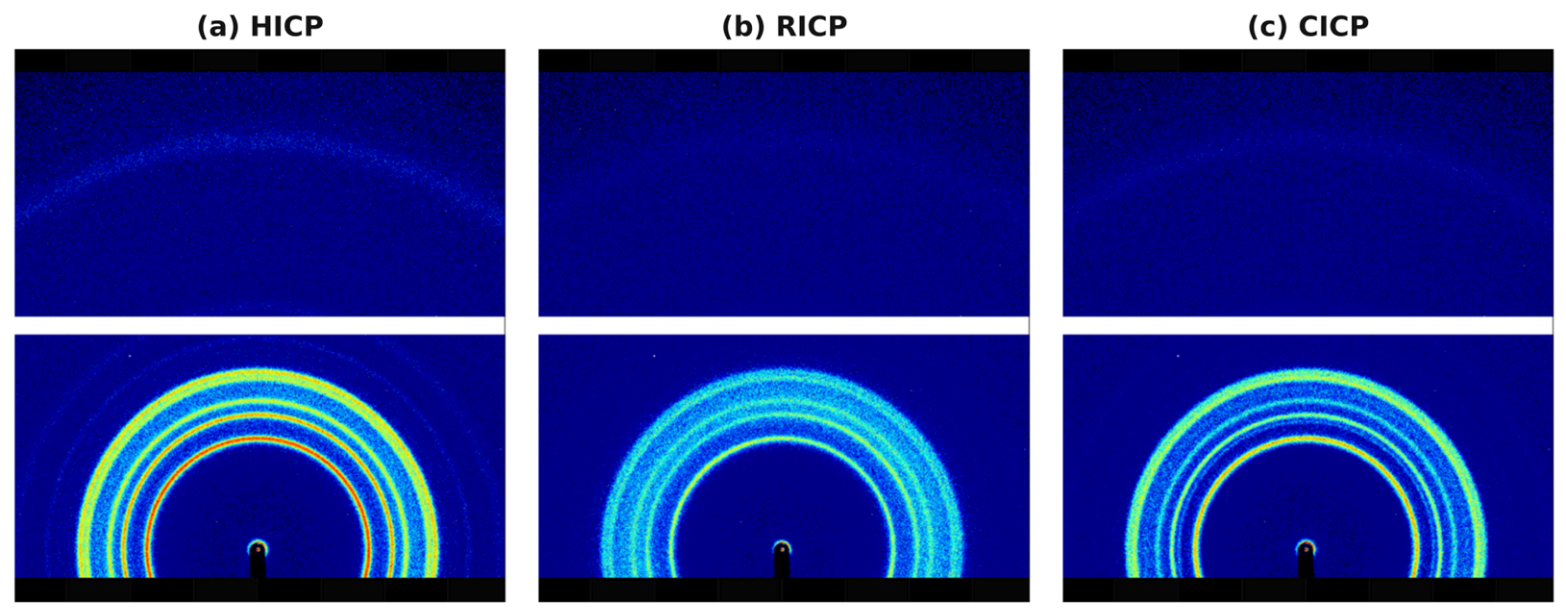
Representative two-dimensional WAXS patterns of (**a**) HICP, (**b**) RICP, and (**c**) CICP.

**Figure 9:**
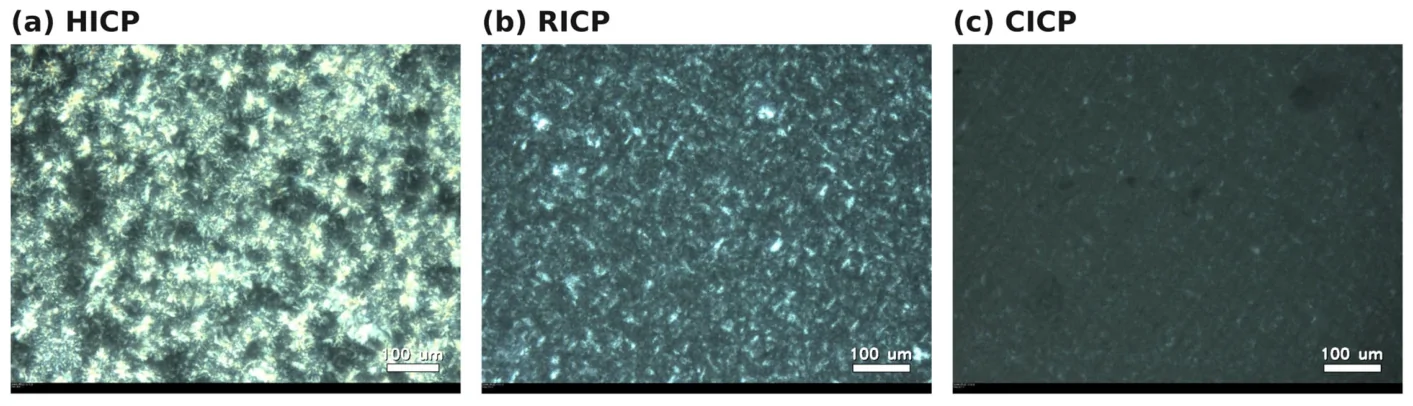
POM images of the crystalline morphology of (**a**) HICP, (**b**) RICP, and (**c**) CICP. All scale bars represent 100 μm.

**Figure 10:**
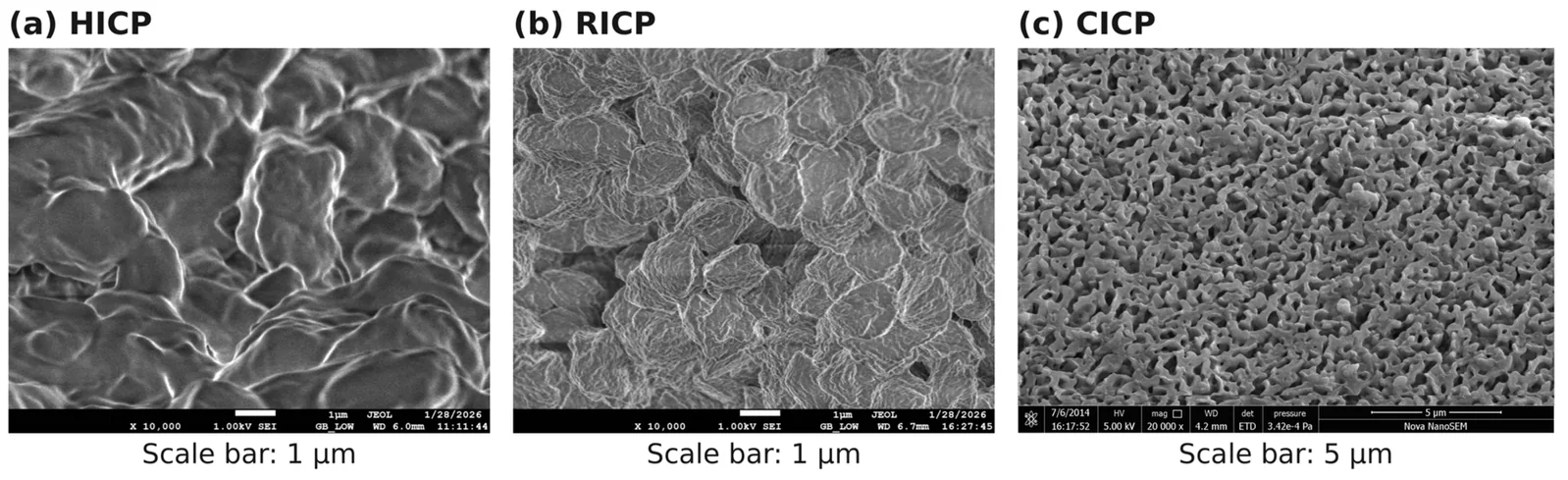
SEM images of cryofractured surfaces after selective xylene etching: (**a**) HICP, (**b**) RICP, and (**c**) CICP. HICP and RICP underwent the same stepwise etching treatment, whereas CICP was etched at room temperature for 1 h. Scale bars represent 1 μm in (**a**,**b**) and 5 μm in (**c**).

**Figure 11:**
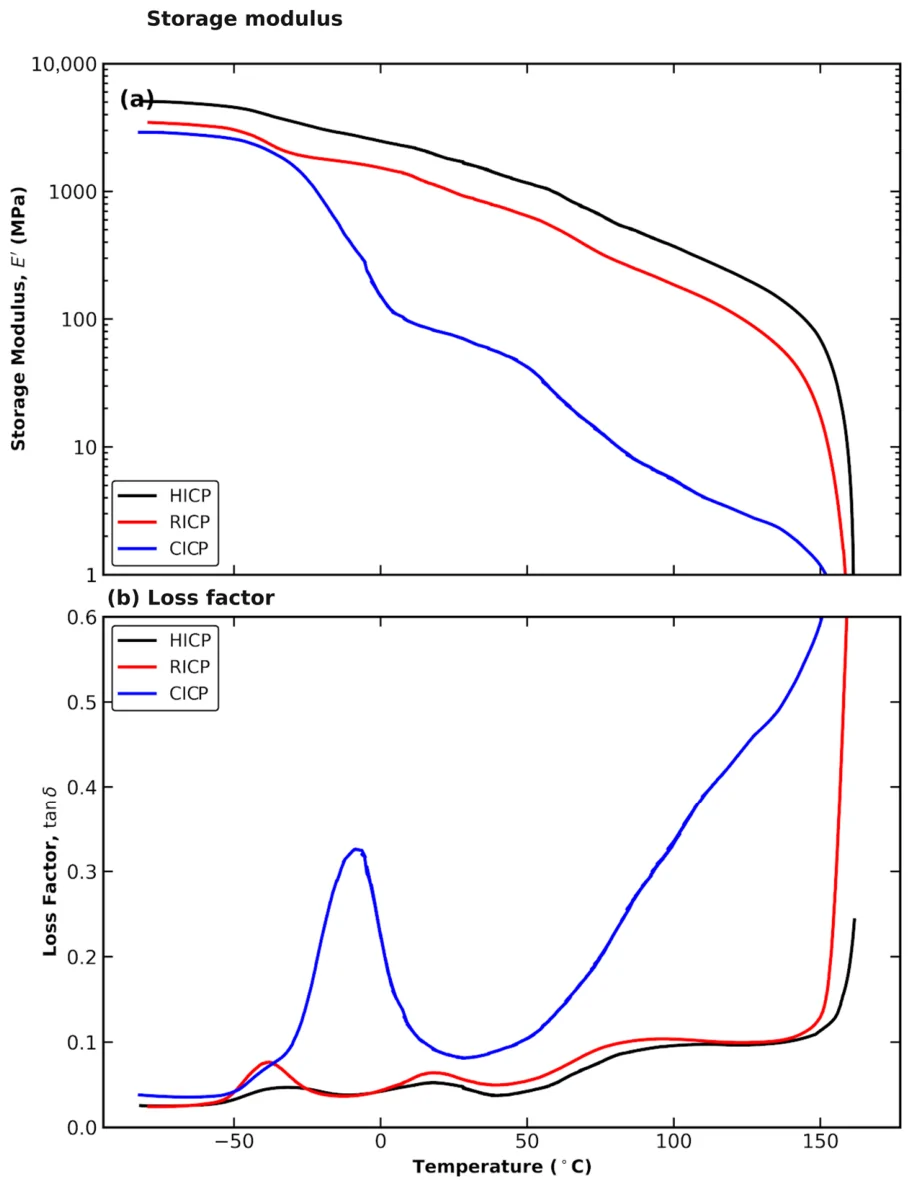
Representative DMA temperature spectra of HICP, RICP, and CICP: (**a**) storage modulus and (**b**) loss factor.

**Figure 12:**
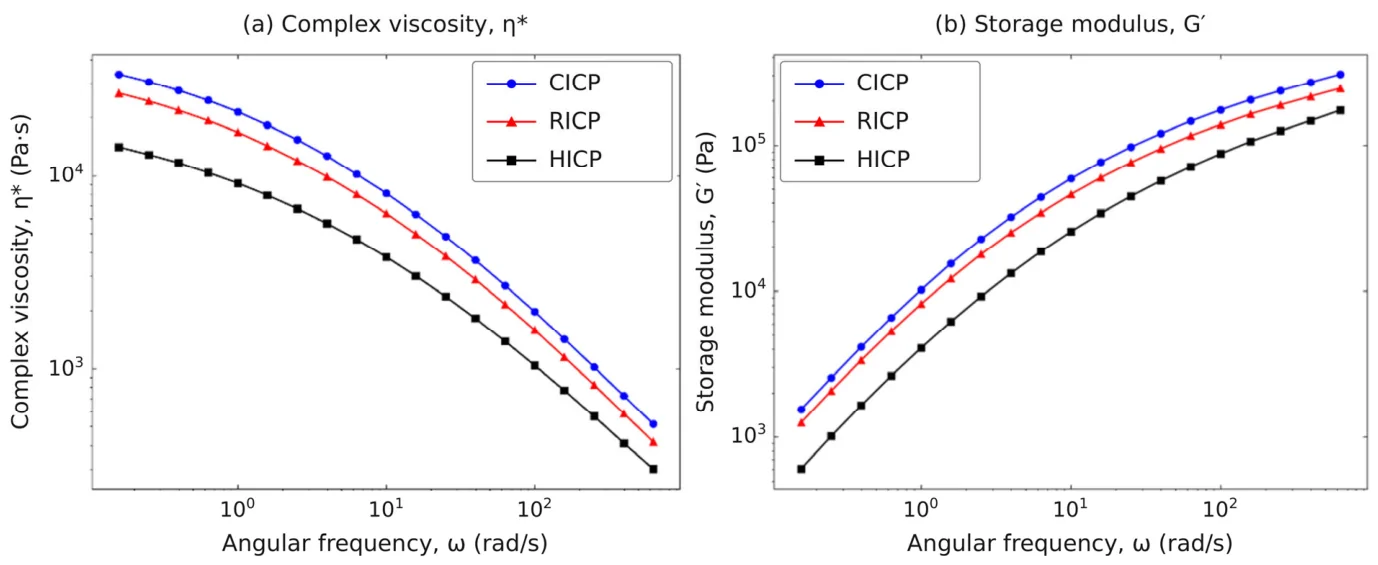
Representative dynamic rheological curves of HICP, RICP, and CICP at 200 °C: (**a**) complex viscosity (η*) and (**b**) storage modulus (G′).

**Figure 13:**
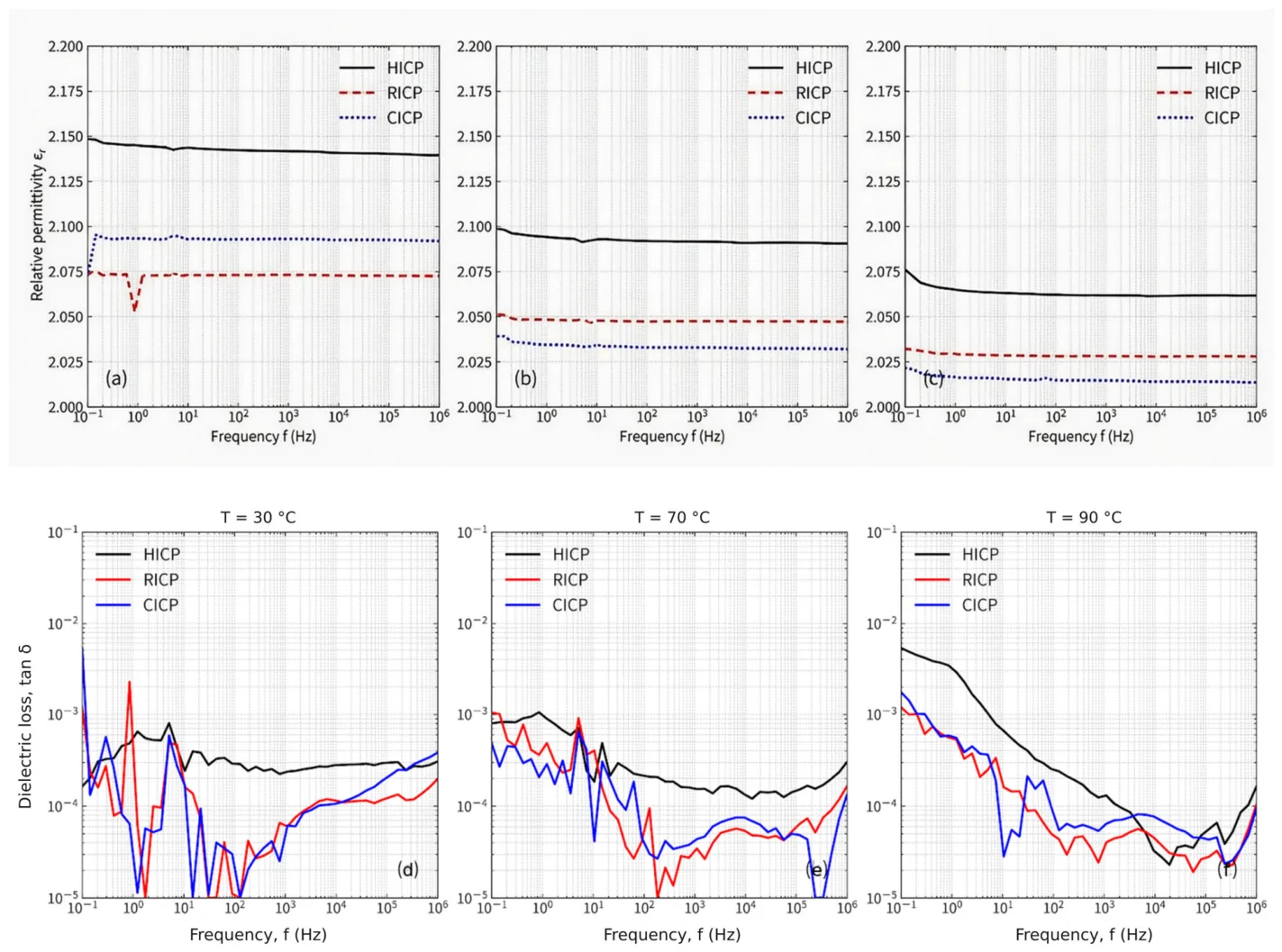
Representative broadband dielectric spectra of HICP, RICP, and CICP: (**a**–**c**) relative permittivity and (**d**–**f**) dielectric loss. Panels (**a**,**d**), (**b**,**e**), and (**c**,**f**) correspond to 30, 70, and 90 °C, respectively.

**Figure 14:**
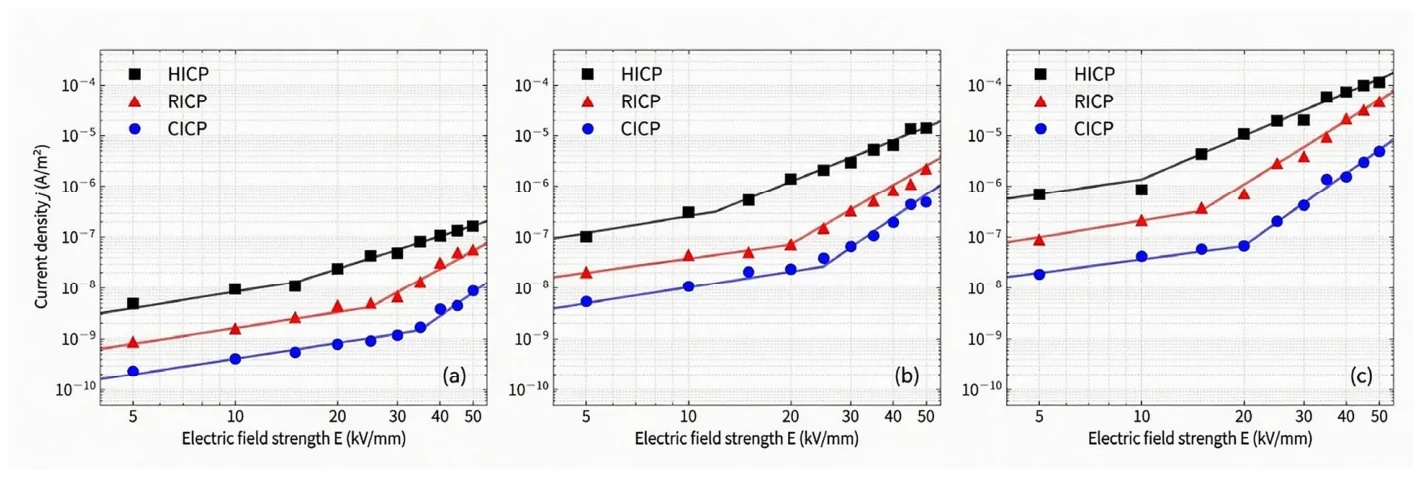
Representative j–E curves of HICP, RICP, and CICP at (**a**) 30, (**b**) 70, and (**c**) 90 °C.

**Figure 15:**
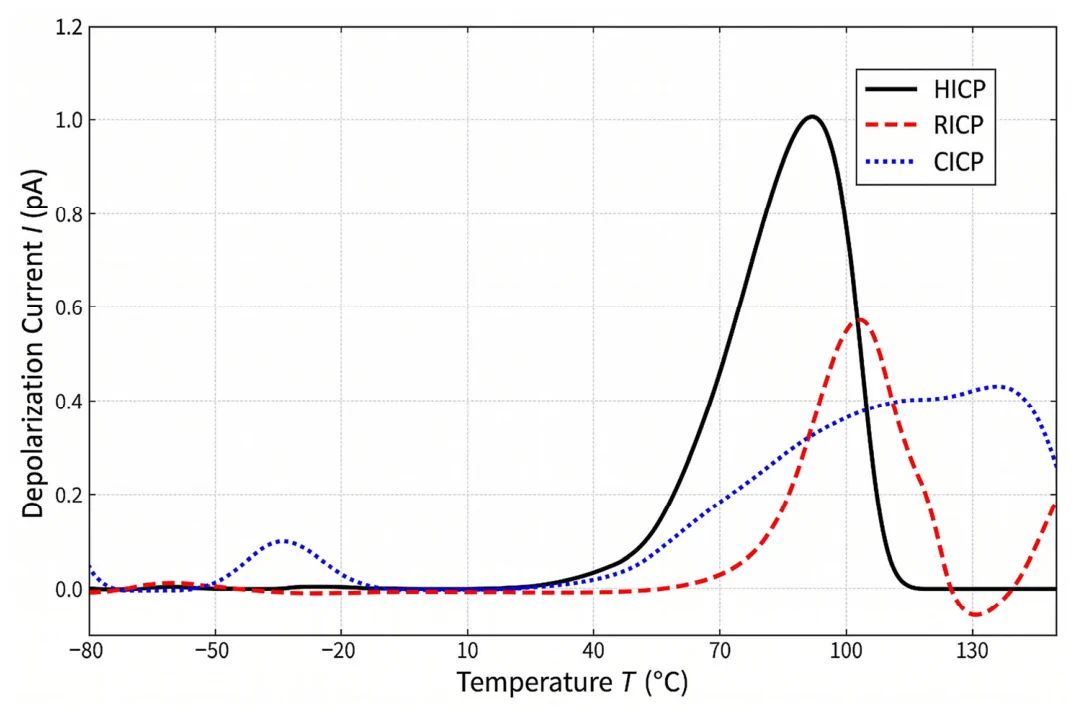
Representative thermally stimulated depolarization current spectra of HICP, RICP, and CICP.

**Figure 16:**
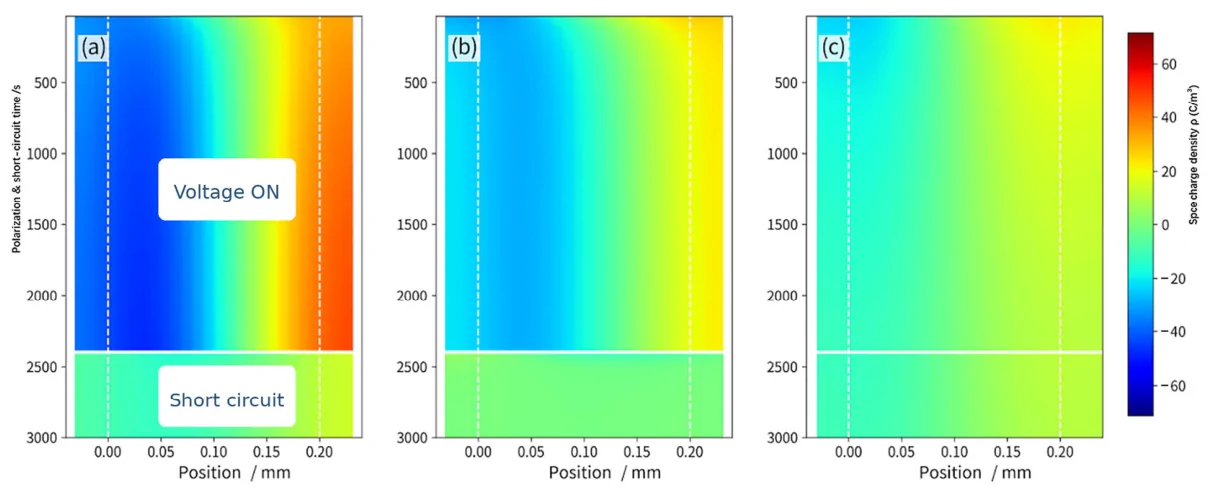
Representative space-charge evolution during DC polarization at 40 kV/mm for 2400 s and subsequent short-circuiting for 600 s: (**a**) HICP, (**b**) RICP, and (**c**) CICP. Vertical white dashed lines mark the specimen thickness, and the horizontal white solid line marks voltage removal at 2400 s.

**Figure 17:**
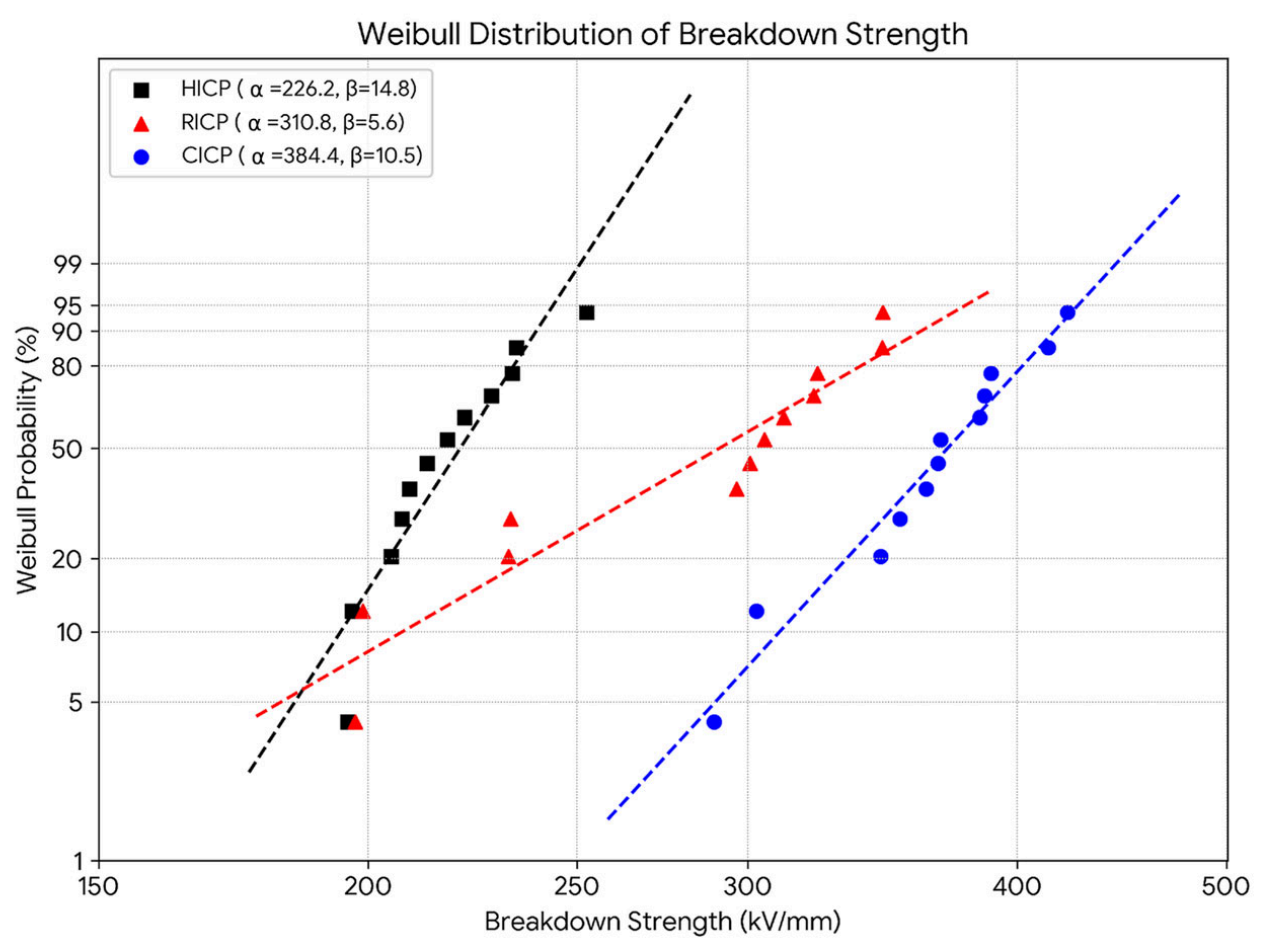
Weibull distributions of DC breakdown strength for HICP, RICP, and CICP. The horizontal axis is logarithmic, and the vertical axis uses a Weibull cumulative failure probability scale. Symbols represent individual specimen breakdown results, and dashed lines represent two-parameter Weibull fits.

**Figure 18:**
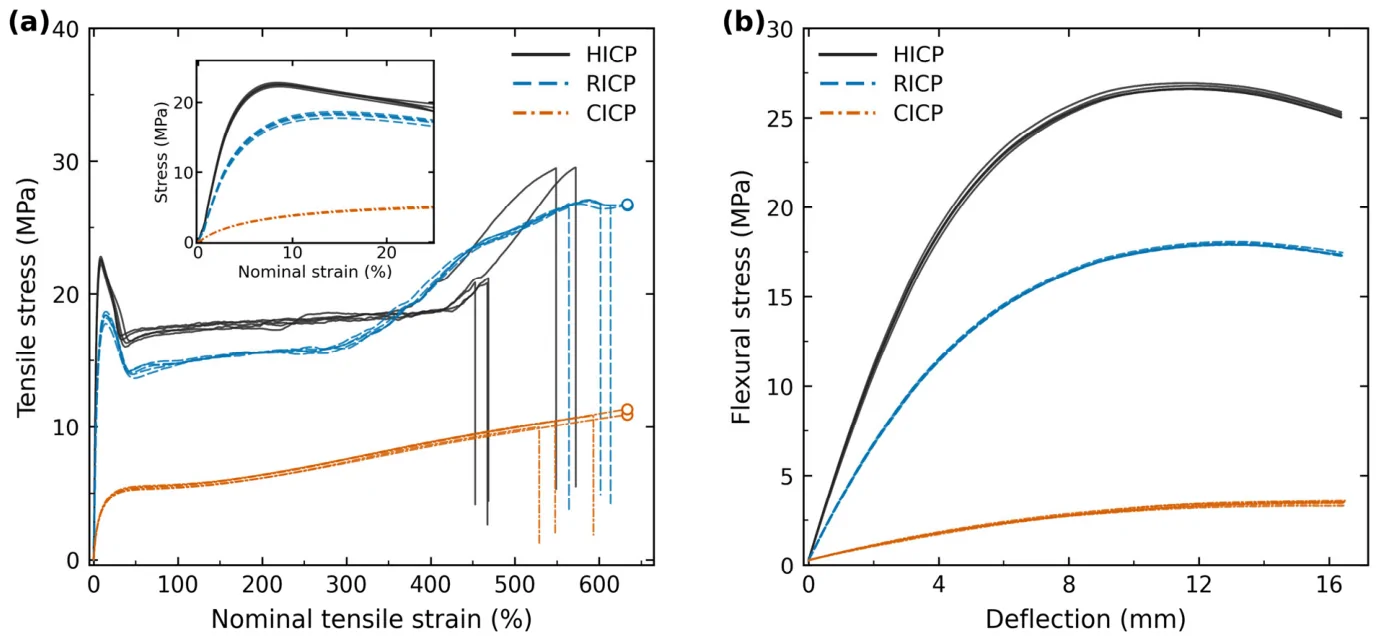
Mechanical responses of HICP, RICP, and CICP: (**a**) tensile stress–nominal strain curves, with an inset showing the low-strain region, and (**b**) flexural stress–deflection curves. Curves of the same color represent the displacement-loading stages of five specimens. Open circles indicate the end of displacement loading.

**Figure 19:**
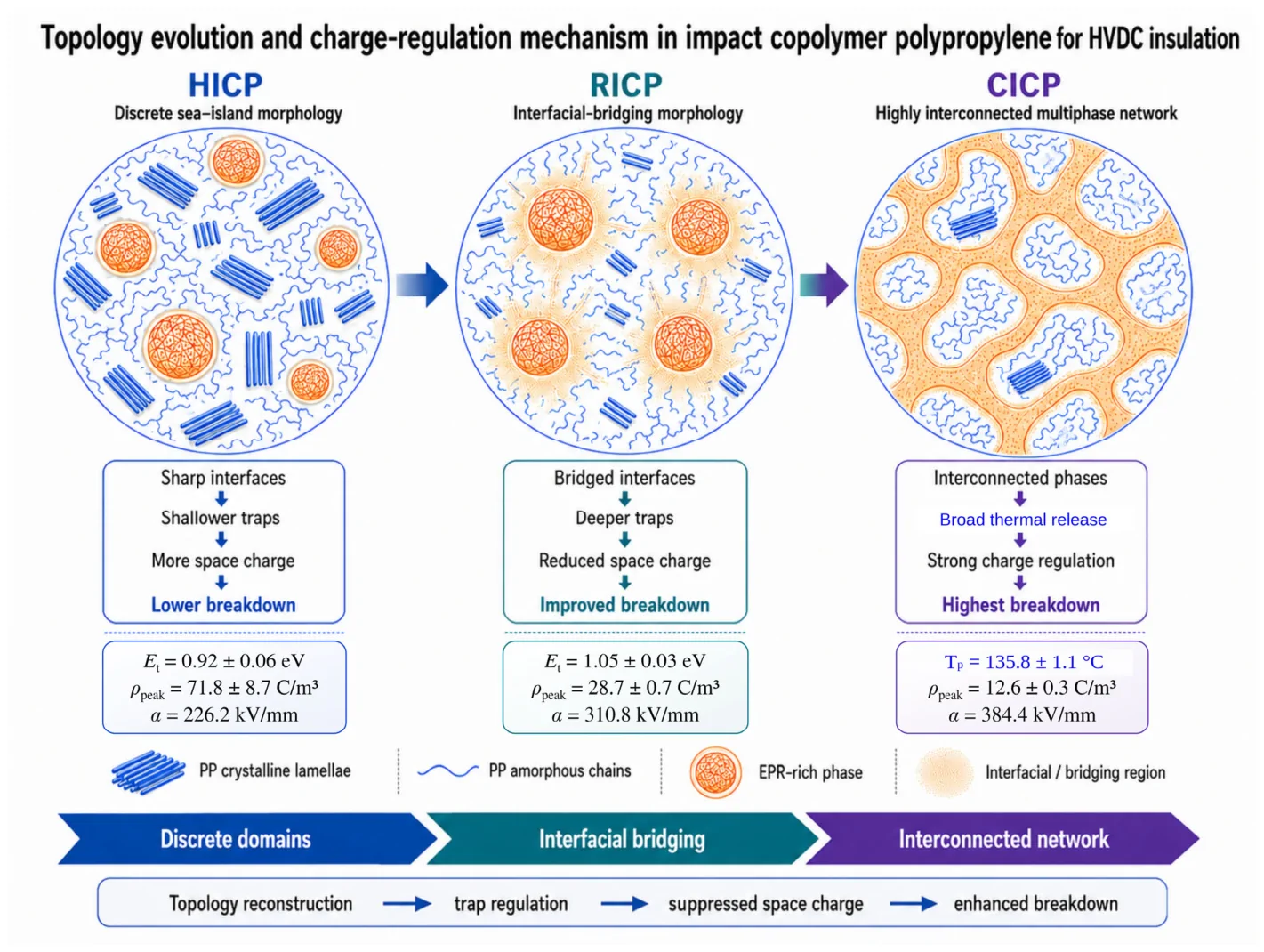
Schematic of ICP multiphase organization and its effects on charge regulation and DC breakdown performance. Characteristic trap depth *E_t_* is shown for HICP and RICP, whereas the high-temperature main-peak temperature *T_p_* is shown for CICP. *ρ*_peak_ is the local peak net-charge density after polarization at 40 kV/mm for 2400 s at room temperature. *E_t_*, *T_p_*, and *ρ*_peak_ are expressed as means ± standard deviations (*n* = 3); α is the Weibull characteristic breakdown strength.

**Table 1:** Specifications, sources, and uses of the experimental reagents.

Reagent	Grade	Source	Use
1,2,4-Trichlorobenzene	Chromatographic grade	Sinopharm Chemical Reagent Co., Ltd. (Shanghai, China)	HT-GPC-IR and CFC/TREF
Deuterated 1,2,4-trichlorobenzene	NMR grade	Sigma-Aldrich Inc. (St. Louis, MO, USA); product 491063	^13^C NMR solvent
Xylene	Analytical grade, ≥99.0%	Sinopharm Chemical Reagent Co., Ltd. (Shanghai, China)	Selective etching for SEM
High-purity nitrogen	≥99.999%	Beijing Dongfang Medical Gas Co., Ltd. (Beijing, China)	Purging for polymerization and DSC purge gas
Silicone oil	Electrical insulating grade	Dow (Midland, MI, USA); XIAMETER PMX-561 Transformer Liquid	DC breakdown test medium

**Table 2:** Total ethylene contents and within-group statistics for HICP, RICP, and CICP.

Sample	Measurement 1 ^a^	Measurement 2	Measurement 3	Mean ± SD
HICP	11.5	11.0	11.8	11.4 ± 0.4
RICP	13.6	14.1	13.4	13.7 ± 0.4
CICP	23.3	23.1	23.4	23.3 ± 0.2

Total ethylene content is expressed in wt%. Superscript a identifies the measurement corresponding to the representative spectrum in Figure 2. Summary values are mean ± sample standard deviation (*n* = 3).

**Table 3:** Independent HT-GPC-IR measurements and within-group statistics for HICP, RICP, and CICP.

Parameter	Sample	Measurement 1 ^a^	Measurement 2	Measurement 3	Mean ± SD
${\bar{M}}_{n}$/(kg·mol^−1^)	HICP	34.1	30.9	35.8	33.6 ± 2.5
${\bar{M}}_{n}$/(kg·mol^−1^)	RICP	38.4	35.0	40.1	37.8 ± 2.6
${\bar{M}}_{n}$/(kg·mol^−1^)	CICP	37.5	35.3	38.2	37.0 ± 1.5
${\bar{M}}_{w}$/(kg·mol^−1^)	HICP	334.4	318.7	337.2	330.1 ± 10.0
${\bar{M}}_{w}$/(kg·mol^−1^)	RICP	411.8	382.9	405.6	400.1 ± 15.2 *
${\bar{M}}_{w}$/(kg·mol^−1^)	CICP	314.6	303.6	314.9	311.0 ± 6.4
*Đ_M_*	HICP	9.81	10.31	9.42	9.85 ± 0.45
*Đ_M_*	RICP	10.72	10.94	10.11	10.59 ± 0.43
*Đ_M_*	CICP	8.39	8.60	8.24	8.41 ± 0.18

Superscript a identifies the measurement corresponding to the representative curve in Figure 3. *Đ_M_* was calculated as ${\bar{M}}_{w}$/${\bar{M}}_{n}$ for each measurement before the results were summarized; *n* = 3. * *p* = 0.0044 versus HICP (two-sided Welch’s *t*-test).

**Table 4:** TREF results and within-group statistics for HICP, RICP, and CICP.

Parameter	Sample	Measurement 1 ^a^	Measurement 2	Measurement 3	Mean ± SD
SF content (T_e_ ≤ 35 °C)/wt%	HICP	18.92	17.46	19.31	18.56 ± 0.98
SF content (T_e_ ≤ 35 °C)/wt%	RICP	27.21	25.83	28.74	27.26 ± 1.46
SF content (T_e_ ≤ 35 °C)/wt%	CICP	73.49	72.64	73.81	73.31 ± 0.60
Matrix elution peak temperature/°C	HICP	125.0	123.9	125.7	124.9 ± 0.9
Matrix elution peak temperature/°C	RICP	112.0	110.8	113.5	112.1 ± 1.4
Matrix elution peak temperature/°C	CICP	123.0	122.6	123.2	122.9 ± 0.3

Superscript a identifies the measurement corresponding to the representative curve in Figure 4. Summary values are means ± sample standard deviations (*n* = 3).

**Table 5:** Melting and crystallization parameters of HICP, RICP, and CICP.

Parameter	Sample	Measurement 1 ^a^	Measurement 2	Measurement 3	Mean ± SD
*T_m_*/°C	HICP	166.4	167.3	165.7	166.5 ± 0.8
*T_m_*/°C	RICP	147.2	146.6	146.5	146.8 ± 0.4
*T_m_*/°C	CICP	164.6	164.9	164.6	164.7 ± 0.2
*T_c_*/°C	HICP	116.4	116.5	116.6	116.5 ± 0.1
*T_c_*/°C	RICP	107.0	107.2	107.9	107.4 ± 0.5
*T_c_*/°C	CICP	108.8	108.5	108.3	108.5 ± 0.3
Δ*H_m_*/(J/g)	HICP	105.8	107.0	109.5	107.4 ± 1.9
Δ*H_m_*/(J/g)	RICP	66.5	66.4	63.8	65.6 ± 1.5
Δ*H_m_*/(J/g)	CICP	28.3	28.0	26.4	27.6 ± 1.0
*X_c_*/%	HICP	51.1	51.7	52.9	51.9 ± 0.9
*X_c_*/%	RICP	32.1	32.1	30.8	31.7 ± 0.7
*X_c_*/%	CICP	13.7	13.5	12.8	13.3 ± 0.5

Superscript a identifies the measurement corresponding to the representative curve in Figure 6. *X_c_* was calculated from Δ*H_m_* for each measurement, and the statistics were calculated before rounding the converted values; *n* = 3.

**Table 6:** Combined WAXS/SAXS/DSC structural parameters of HICP, RICP, and CICP.

Parameter	Sample	Measurement 1 ^a^	Measurement 2	Measurement 3	Mean ± SD
*D*_110_/nm	HICP	13.87	15.19	14.29	14.45 ± 0.67
*D*_110_/nm	RICP	11.75	11.47	12.52	11.91 ± 0.54
*D*_110_/nm	CICP	12.44	12.60	12.11	12.38 ± 0.25
*L_p_*/nm	HICP	15.71	15.06	15.86	15.54 ± 0.43
*L_p_*/nm	RICP	14.61	15.05	14.53	14.73 ± 0.28
*L_p_*/nm	CICP	14.28	14.24	14.35	14.29 ± 0.06
*φ_c_*/%	HICP	48.7	49.3	50.5	49.5 ± 0.9
*φ_c_*/%	RICP	30.1	30.0	28.8	29.6 ± 0.7
*φ_c_*/%	CICP	12.6	12.4	11.7	12.2 ± 0.5
*d_c_*/nm	HICP	7.65	7.42	8.01	7.69 ± 0.30
*d_c_*/nm	RICP	4.39	4.52	4.19	4.36 ± 0.17
*d_c_*/nm	CICP	1.80	1.77	1.68	1.75 ± 0.06
*d_a_*/nm	HICP	8.06	7.64	7.85	7.85 ± 0.21
*d_a_*/nm	RICP	10.22	10.53	10.34	10.37 ± 0.16
*d_a_*/nm	CICP	12.48	12.47	12.67	12.54 ± 0.11
SAXS FWHM/nm^−1^	HICP	0.229	0.223	0.228	0.227 ± 0.003
SAXS FWHM/nm^−1^	RICP	0.310	0.301	0.319	0.310 ± 0.009
SAXS FWHM/nm^−1^	CICP	0.294	0.292	0.300	0.295 ± 0.004

Superscript a identifies the representative results corresponding to Figure 7 and Figure 8; *n* = 3. *X_c_* is the apparent PP crystallinity on a whole-sample mass basis. *φ_c_* was calculated from unrounded *X_c_* values; *d_c_* and *d_a_* were calculated for each paired *L_p_* value and the correspondingly numbered *X_c_* value in Table 5 before summarization. *φ_c_* is the crystalline volume fraction in the two-phase reference model. *d_c_* and *d_a_* are the combined quantities corresponding to the crystalline and amorphous reference fractions, respectively. FWHM denotes the full width at half maximum of the main Lorentz-corrected SAXS peak.

**Table 7:** Mean film thickness and haze of HICP, RICP, and CICP.

Sample	Mean Thickness/mm	Film 1	Film 2	Film 3	Film 4	Film 5	Mean Haze ± SD/%
HICP	0.214	97.3	97.9	98.3	96.5	98.4	97.7 ± 0.8
RICP	0.221	78.3	77.4	78.2	78.0	76.6	77.7 ± 0.7
CICP	0.201	64.7	65.2	63.5	64.2	65.2	64.6 ± 0.7

Haze is expressed as a percentage. The haze of each film is the mean of measurements at three positions. Group means and sample standard deviations were calculated from the mean haze values of five individual films (*n* = 5).

**Table 8:** Characteristic DMA parameters of HICP, RICP, and CICP.

Parameter	Sample	Measurement 1 ^a^	Measurement 2	Measurement 3	Mean ± SD
E′ at −50 °C (MPa)	HICP	4559	4625	4274	4490 ± 190
E′ at −50 °C (MPa)	RICP	3043	3061	3082	3062 ± 20
E′ at −50 °C (MPa)	CICP	2574	2518	2561	2551 ± 29
E′ at 25 °C (MPa)	HICP	1760	1791	1664	1738 ± 66
E′ at 25 °C (MPa)	RICP	983	949	977	970 ± 18
E′ at 25 °C (MPa)	CICP	74.9	74.2	74.7	74.6 ± 0.4
E′ at 100 °C (MPa)	HICP	372	387	346	368 ± 21
E′ at 100 °C (MPa)	RICP	186	182	201	190 ± 10
E′ at 100 °C (MPa)	CICP	5.4	5.4	5.6	5.5 ± 0.1
Rubber-rich phase peak temperature (°C)	HICP	−31.2	−31.7	−31.4	−31.4 ± 0.3
Rubber-rich phase peak temperature (°C)	RICP	−38.0	−39.7	−37.0	−38.2 ± 1.4
Rubber-rich phase shoulder temperature (°C)	CICP	−36.2	−36.2	−33.1	−35.2 ± 1.8
Amorphous PP matrix peak temperature (°C)	HICP	18.3	18.8	18.9	18.7 ± 0.3
Amorphous PP matrix peak temperature (°C)	RICP	17.9	16.6	18.4	17.6 ± 0.9
Main peak temperature of overlapping low-temperature relaxations (°C)	CICP	−8.4	−7.8	−8.4	−8.2 ± 0.3
α* relaxation peak temperature (°C)	HICP	111.4	110.2	113.5	111.7 ± 1.7
α* relaxation peak temperature (°C)	RICP	96.0	94.1	97.5	95.9 ± 1.7
α* relaxation peak temperature (°C)	CICP	—	—	—	—

Superscript a identifies the representative curve in Figure 11; *n* = 3. The two low-temperature features of CICP are the shoulder and the main peak in the overlapping relaxation region, respectively; its α* peak temperature is indicated by an em dash.

**Table 9:** Characteristic dynamic rheological parameters of HICP, RICP, and CICP at 200 °C.

Parameter	Sample	Measurement 1 ^a^	Measurement 2	Measurement 3	Mean ± SD
η* (ω = 1 rad/s)/(kPa·s)	HICP	9.1	8.7	9.3	9.0 ± 0.3
η* (ω = 1 rad/s)/(kPa·s)	RICP	16.7	17.3	14.9	16.3 ± 1.2
η* (ω = 1 rad/s)/(kPa·s)	CICP	21.2	22.4	20.6	21.4 ± 0.9
G′ (ω = 1 rad/s)/kPa	HICP	4.1	4.0	4.4	4.2 ± 0.2
G′ (ω = 1 rad/s)/kPa	RICP	8.2	8.5	7.2	8.0 ± 0.7
G′ (ω = 1 rad/s)/kPa	CICP	10.2	10.8	10.0	10.3 ± 0.4
η* (ω = 100 rad/s)/(kPa·s)	HICP	1.05	1.04	1.05	1.05 ± 0.01
η* (ω = 100 rad/s)/(kPa·s)	RICP	1.60	1.66	1.44	1.57 ± 0.11
η* (ω = 100 rad/s)/(kPa·s)	CICP	1.98	2.04	1.92	1.98 ± 0.06
G′ (ω = 100 rad/s)/kPa	HICP	88	88	87	88 ± 1
G′ (ω = 100 rad/s)/kPa	RICP	138	144	124	135 ± 10
G′ (ω = 100 rad/s)/kPa	CICP	175	181	169	175 ± 6

Superscript a denotes approximate readings from the representative curves in Figure 12 at the corresponding frequencies. Summary values are means ± sample standard deviations; *n* = 3. η* denotes complex viscosity.

**Table 10:** Characteristic broadband dielectric parameters of HICP, RICP, and CICP.

Parameter and Conditions	Material	Measurement 1 ^a^	Measurement 2	Measurement 3	Mean ± SD
ε′30 °C, 1 kHz	HICP	2.142	2.141	2.131	2.138 ± 0.006
ε′30 °C, 1 kHz	RICP	2.073	2.096	2.086	2.085 ± 0.012
ε′30 °C, 1 kHz	CICP	2.093	2.099	2.097	2.096 ± 0.003
ε′70 °C, 1 kHz	HICP	2.092	2.092	2.085	2.090 ± 0.004
ε′70 °C, 1 kHz	RICP	2.047	2.072	2.061	2.060 ± 0.013
ε′70 °C, 1 kHz	CICP	2.033	2.042	2.036	2.037 ± 0.005
ε′90 °C, 1 kHz	HICP	2.062	2.065	2.053	2.060 ± 0.006
ε′90 °C, 1 kHz	RICP	2.028	2.047	2.043	2.039 ± 0.010
ε′90 °C, 1 kHz	CICP	2.014	2.021	2.016	2.017 ± 0.004
tan δ (×10^−3^) 90 °C, 0.1 Hz	HICP	5.3	4.7	5.4	5.1 ± 0.4
tan δ (×10^−3^) 90 °C, 0.1 Hz	RICP	1.2	1.2	1.1	1.2 ± 0.1
tan δ (×10^−3^) 90 °C, 0.1 Hz	CICP	1.7	1.7	1.6	1.7 ± 0.1

Superscript a denotes approximate readings from the representative curves in Figure 13 at the corresponding temperature and frequency. The sample size for each row is 3.

**Table 11:** Characteristic high-field conduction parameters of HICP, RICP, and CICP at different temperatures.

Parameter and Conditions	Material	Measurement 1 ^a^	Measurement 2	Measurement 3	Mean ± SD
Characteristic transition field at 30 °C (kV/mm)	HICP	14.8	14.8	13.7	14.4 ± 0.6
Characteristic transition field at 30 °C (kV/mm)	RICP	24.3	27.1	24.9	25.4 ± 1.5
Characteristic transition field at 30 °C (kV/mm)	CICP	34.2	35.7	31.7	33.9 ± 2.0
Characteristic transition field at 70 °C (kV/mm)	HICP	11.6	11.5	11.0	11.4 ± 0.3
Characteristic transition field at 70 °C (kV/mm)	RICP	19.5	21.9	19.9	20.4 ± 1.3
Characteristic transition field at 70 °C (kV/mm)	CICP	25.4	26.1	24.0	25.2 ± 1.1
Characteristic transition field at 90 °C (kV/mm)	HICP	9.7	9.4	9.3	9.5 ± 0.2
Characteristic transition field at 90 °C (kV/mm)	RICP	15.6	17.4	15.4	16.1 ± 1.1
Characteristic transition field at 90 °C (kV/mm)	CICP	19.1	19.8	18.4	19.1 ± 0.7
Current density j at 30 °C and 50 kV/mm (A/m^2^)	HICP	1.5 × 10^−7^	1.2 × 10^−7^	1.5 × 10^−7^	(1.4 ± 0.2) × 10^−7^
Current density j at 30 °C and 50 kV/mm (A/m^2^)	RICP	5.5 × 10^−8^	5.6 × 10^−8^	4.7 × 10^−8^	(5.3 ± 0.5) × 10^−8^
Current density j at 30 °C and 50 kV/mm (A/m^2^)	CICP	9.5 × 10^−9^	8.9 × 10^−9^	7.7 × 10^−9^	(8.7 ± 0.9) × 10^−9^

Superscript a identifies the characteristic parameters corresponding to the representative curves in Figure 14. The sample size for each row is 3.

**Table 12:** Characteristic TSDC and space-charge parameters of HICP, RICP, and CICP.

Parameter and Conditions	Material	Measurement 1 ^a^	Measurement 2	Measurement 3	Mean ± SD
Main peak temperature *T_p_* (°C)	HICP	92.5	93.1	90.4	92.0 ± 1.4
Main peak temperature *T_p_* (°C)	RICP	105.0	107.4	103.4	105.3 ± 2.0
Main peak temperature *T_p_* (°C)	CICP	136.6	136.3	134.6	135.8 ± 1.1
Characteristic trap depth *E_t_* (eV)	HICP	0.92	0.98	0.87	0.92 ± 0.06
Characteristic trap depth *E_t_* (eV)	RICP	1.05	1.03	1.08	1.05 ± 0.03
Characteristic trap depth *E_t_* (eV)	CICP	—	—	—	—
Equivalent thermally released carrier number density *N_t_* (10^16^ m^−3^)	HICP	2.84	3.47	2.77	3.03 ± 0.39
Equivalent thermally released carrier number density *N_t_* (10^16^ m^−3^)	RICP	1.35	1.79	1.20	1.45 ± 0.31
Equivalent thermally released carrier number density *N_t_* (10^16^ m^−3^)	CICP	2.69	2.53	2.31	2.51 ± 0.19
Local peak charge density at room temperature, 40 kV/mm, 2400 s (C/m^3^)	HICP	65.5	68.2	81.7	71.8 ± 8.7
Local peak charge density at room temperature, 40 kV/mm, 2400 s (C/m^3^)	RICP	28.4	29.5	28.2	28.7 ± 0.7
Local peak charge density at room temperature, 40 kV/mm, 2400 s (C/m^3^)	CICP	12.5	12.9	12.4	12.6 ± 0.3

Data are means ± sample standard deviations (*n* = 3). Superscript a for the TSDC and PEA parameters identifies the representative results in Figure 15 and Figure 16, respectively. The em dash in the *Et row* for CICP indicates that the complete full width at half maximum of its high-temperature peak was not obtained within the tested temperature range. Peak temperature, equivalent number density of thermally released carriers, and peak PEA charge density were summarized separately.

**Table 13:** Weibull parameters for DC breakdown of HICP, RICP, and CICP.

Material	Characteristic Breakdown Strength α (kV/mm)	Shape Parameter β
HICP	226.2	14.8
RICP	310.8	5.6
CICP	384.4	10.5

**Table 14:** Macroscopic mechanical properties of HICP, RICP, and CICP.

Property	Unit	HICP	RICP	CICP
Tensile strength/maximum tensile stress	MPa	22.5 ± 0.2	18.3 ± 0.3	10.7 ± 0.5
Tensile elastic modulus	MPa	1000 ± 40	612 ± 22	89.4 ± 4.9
Flexural modulus	MPa	962 ± 31	590 ± 10	72.8 ± 3.6
Flexural stress at specified deflection	MPa	23.4 ± 0.2	14.8 ± 0.1	2.39 ± 0.04
Notched impact strength (23 °C)	kJ/m^2^	46 ± 1 (P)	68 ± 4 (P)	35 ± 2 (P)
Notched impact strength (0 °C)	kJ/m^2^	9.0 ± 0.2 (C)	9.3 ± 0.3 (C)	56 ± 3 (P)
Notched impact strength (−20 °C)	kJ/m^2^	6.4 ± 0.1 (C)	5.9 ± 0.2 (C)	97 ± 4 (P)

Data are means ± standard deviations. The number of specimens for tensile and flexural tests was 5, and that for impact tests was 10. P denotes partial break, and C denotes complete break. The first local stress maximum is reported for HICP and RICP; the maximum tensile stress during displacement loading is reported for CICP.

## Data Availability

The data that support the findings of this study are available from the corresponding author upon reasonable request.

## References

[1] Hosier I.L., Vaughan A.S., Swingler S.G. (2011). An investigation of the potential of polypropylene and its blends for use in recyclable high voltage cable insulation systems. J. Mater. Sci..

[2] Ouyang Y., Pourrahimi A.M., Östergren I., Mellqvist M., Ånevall J., Soroudi A., Lund A., Xu X., Gkourmpis T., Hagstrand P.-O. (2022). Highly insulating thermoplastic blends comprising a styrenic copolymer for direct-current power cable insulation. High Volt..

[3] Wu J., Dang B., Hu J. (2020). Comparison of Effects of Ethylene-Based and Propylene-Based Copolymer on Tailoring the Properties of Polypropylene. IEEE Access.

[4] Gahleitner M., Jääskeläinen P., Ratajski E., Paulik C., Reussner J., Wolfschwenger J., Neißl W. (2005). Propylene–ethylene random copolymers: Comonomer effects on crystallinity and application properties. J. Appl. Polym. Sci..

[5] Gao Y., Li J., Yuan Y., Huang S., Du B. (2018). Trap Distribution and Dielectric Breakdown of Isotactic Polypropylene/Propylene Based Elastomer With Improved Flexibility for DC Cable Insulation. IEEE Access.

[6] Yang K., Liu Y., Yan Z., Tian Y., Liu Y., Jing Z., Li J., Li S. (2020). Enhanced Morphology-Dependent Tensile Property and Breakdown Strength of Impact Polypropylene Copolymer for Cable Insulation. Materials.

[7] van Reenen A.J., Basson N.C. (2012). Molecular composition and properties of impact propylene copolymers. Express Polym. Lett..

[8] Aarnio-Winterhof M., Doshev P., Seppälä J., Gahleitner M. (2017). Structure-property relations of heterophasic ethylene-propylene copolymers based on a single-site catalyst. Express Polym. Lett..

[9] Huang X., Yang J., Zhao X., Yang X., Wang K., Wang D., Fu Z. (2025). Influence of the Heterophasic Structure and Its Characteristics on the DC Electrical Properties of Impact Polypropylene Copolymer. Polymers.

[10] Zhang W., Xu M., Huang K., Mu Q., Chen G. (2019). Effect of β-crystals on the mechanical and electrical properties of β-nucleated isotactic polypropylene. IEEE Trans. Dielectr. Electr. Insul..

[11] Zhan Y., Yang X., Yang J., Hou S., Fu M. (2024). Improved Electrical Properties of Organic Modified Thermoplastic Insulation Material for Direct Current Cable Application. Polymers.

[12] Min D., Yan C., Mi R., Ma C., Huang Y., Li S., Wu Q., Xing Z. (2018). Carrier Transport and Molecular Displacement Modulated dc Electrical Breakdown of Polypropylene Nanocomposites. Polymers.

[13] Cancelas A.J., Monteil V., McKenna T.F.L. (2016). Influence of Activation Conditions on the Gas Phase Polymerisation of Propylene. Macromol. Symp..

[14] Liu X., Zhang B., Guo W., Jiang B., Du M., Fu Z., Fan Z. (2025). Hydrogen Responses of Propylene Polymerization with MgCl_2_-Supported Ziegler–Natta Catalysts in the Presence of Different Silane External Donors. Catalysts.

[15] Govindaswamy P., Wada E., Kono H., Uozumi T., Funabashi H. (2022). Propylene Polymerization Performance with Ziegler-Natta Catalysts Combined with U-Donor and T01 Donor as External Donor. Catalysts.

[16] Valaei S., Bartke M. (2022). Multistep Bulk-/Gas-Phase Polymerization of Propene in a Reaction Calorimeter. Macromol. React. Eng..

[17] Dong X., Fan X., Wang W. (2025). Comprehensive Performance Regulation and Characterization of Polypropylene/Elastomer Composite Insulation Materials. Polymers.

[18] Singh G., Kothari A.V., Gupta V.K. (2009). Triad sequence determination of ethylene–propylene copolymers—Application of quantitative ^13^C NMR. Polym. Test..

[19] Giraudeau P., Wang J.L., Baguet É. (2006). Improvement of the inverse-gated-decoupling sequence for a faster quantitative analysis by ^13^C NMR. C. R. Chim..

[20] (2016). Plastics—Determination of Monomer Contents and Sequential Structure of Polypropylene Copolymerized with Ethylene—Carbon-13 Nuclear Magnetic Resonance.

[21] Di Martino S., Kelchtermans M. (1995). Determination of the composition of ethylene–propylene–rubbers using ^13^C-NMR spectroscopy. J. Appl. Polym. Sci..

[22] Wang X., Hu S., Guo Y., Li G., Xu R. (2019). Toughened High-Flow Polypropylene with Polyolefin-Based Elastomers. Polymers.

[23] (2021). Standard Test Method for Transition Temperatures and Enthalpies of Fusion and Crystallization of Polymers by Differential Scanning Calorimetry.

[24] Gee D.R., Melia T.P. (1970). Thermal properties of melt and solution crystallized isotactic polypropylene. Makromol. Chem..

[25] Patterson A.L. (1939). The Scherrer Formula for X-Ray Particle Size Determination. Phys. Rev..

[26] Ryan A.J., Bras W., Mant G.R., Derbyshire G.E. (1994). A direct method to determine the degree of crystallinity and lamellar thickness of polymers: Application to polyethylene. Polymer.

[27] Sangroniz L., Carmeli E., Vukusic L., Hristov V., Galatini M., Tranchida D., Cavallo D. (2025). Lamellar thickness of the polypropylene matrix determines surface induced nucleation of polyethylene droplets in immiscible blends. Polymer.

[28] Fratini C.M. (2006). Study of the Morphology and Optical Properties of Propylene/Ethylene Copolymer Films. Ph.D. Thesis.

[29] (2023). Dielectric and Resistive Properties of Solid Insulating Materials—Part 3-1: Determination of Resistive Properties (DC Methods)—Volume Resistance and Volume Resistivity—General Method.

[30] Li H., Gadinski M.R., Huang Y., Ren L., Zhou Y., Ai D., Han Z., Yao B., Wang Q. (2020). Crosslinked fluoropolymers exhibiting superior high-temperature energy density and charge–discharge efficiency. Energy Environ. Sci..

[31] Maeno T. (1999). Calibration of the pulsed electroacoustic method for measuring space charge density. IEEJ Trans. Fundam. Mater..

[32] (2013). Electric Strength of Insulating Materials—Test Methods—Part 1: Tests at Power Frequencies.

[33] (2013). Electric Strength of Insulating Materials—Test Methods—Part 2: Additional Requirements for Tests Using Direct Voltage.

[34] (2007). Guide for the Statistical Analysis of Electrical Insulation Breakdown Data.

[35] Scholz F.W. (2015). Inference for the Weibull Distribution: A tutorial. Quant. Methods Psychol..

[36] (2022). Plastics—Determination of Tensile Properties—Part 2: Test Conditions for Moulding and Extrusion Plastics.

[37] (2012). Plastics—Determination of Tensile Properties—Part 1: General Principles.

[38] (2008). Plastics—Determination of Flexural Properties.

[39] (2008). Plastics—Determination of Charpy Impact Properties—Part 1: Non-Instrumented Impact Test.

[40] Kolbert A.C., Didier J.G. (1999). Determination of monomer sequence distribution in EPDM by ^13^C-NMR: Third monomer effects. J. Appl. Polym. Sci..

[41] Zhang J.C., Guo M.F., Huang H.H., Tang Y.J., Hou L.P., Sheng J.F., Liu X.B. (2014). The characterization and performance study of propylene/1-butylene and propylene/ethylene random copolymers. Sci. Sin. Chim..

[42] Chen J.W., Dai J., Yang J.H., Huang T., Zhang N., Wang Y. (2013). Annealing-induced crystalline structure and mechanical property changes of polypropylene random copolymer. J. Mater. Res..

[43] Gubała D., Slastanova A., Matthews L., Islas L., Wąsik P., Cacho-Nerin F., Ferreira Sanchez D., Robles E., Chen M., Briscoe W.H. (2024). Effects of Erucamide on Fiber “Softness”: Linking Single-Fiber Crystal Structure and Mechanical Properties. ACS Nano.

[44] Caelers H.J.M., Troisi E.M., Govaert L.E., Peters G.W.M. (2017). Deformation-Induced Phase Transitions in iPP Polymorphs. Polymers.

[45] Ketsamee P., Andritsch T., Vaughan A. An Investigation of Mechanical Properties and Breakdown Strength of Polypropylene/Ultra-High Molecular Weight Polyethylene Nanocomposites. Proceedings of the 2022 IEEE Conference on Electrical Insulation and Dielectric Phenomena (CEIDP).

[46] Akpalu Y.A., Amis E.J. (1999). Evolution of density fluctuations to lamellar crystals in linear polyethylene. J. Chem. Phys..

[47] Molnár J., Sepsi Ö., Erdei G., Lenk S., Ujhelyi F., Menyhárd A. (2020). Modeling of light scattering and haze in semicrystalline polymers. J. Polym. Sci..

[48] Ahmad Z., Kumar K.D., Saroop M., Preschilla N., Biswas A., Bellare J.R., Bhowmick A.K. (2010). Highly transparent thermoplastic elastomer from isotactic polypropylene and styrene/ethylene-butylene/styrene triblock copolymer: Structure-property correlations. Polym. Eng. Sci..

[49] Doshev P., Lohse G., Henning S., Krumova M., Heuvelsland A., Michler G., Radusch H.J. (2006). Phase interactions and structure evolution of heterophasic ethylene–propylene copolymers as a function of system composition. J. Appl. Polym. Sci..

[50] Genovese A., Shanks R.A. (2004). Dynamic Mechanical Properties of Poly(propylene) Blends with Poly[ethylene-co-(methyl acrylate)]. Macromol. Mater. Eng..

[51] Zhang J.Q., Zhang Y.J., Zhang C.Y., Chen Q. (2023). Structural and Rheological Properties of PP/EPR/PE Alloys. Chin. J. Polym. Sci..

[52] Bagheri H., Jahani Y., Nekoomanesh Haghighi M., Hakim S., Fan Z.Q. (2011). Dynamic shear rheological behavior of PP/EPR in-reactor alloys synthesized by multi-stage sequential polymerization process. J. Appl. Polym. Sci..

[53] Grein C., Gahleitner M., Knogler B., Nestelberger S. (2007). Melt viscosity effects in ethylene–propylene copolymers. Rheol. Acta.

[54] Zhang H., Duan Y., Xie M., Ma G., Li P., Qin J. (2022). The effects of TiO_2_ nanoparticles on the temperature-dependent electrical and dielectric properties of polypropylene. Front. Energy Res..

[55] Ruuska H., Arola E., Kannus K., Rantala T.T., Valkealahti S. (2008). Feasibility of density functional methods to predict dielectric properties of polymers. J. Chem. Phys..

[56] Fothergill J.C., Dodd S.J., Dissado L.A., Liu T., Nilsson U.H. (2011). The measurement of very low conductivity and dielectric loss in XLPE cables: A possible method to detect degradation due to thermal aging. IEEE Trans. Dielectr. Electr. Insul..

[57] He X., Rytöluoto I., Anyszka R., Mahtabani A., Saarimäki E., Lahti K., Paajanen M., Dierkes W., Blume A. (2019). Surface Modification of Fumed Silica by Plasma Polymerization of Acetylene for PP/POE Blends Dielectric Nanocomposites. Polymers.

[58] Vu T.T.N., Teyssedre G., Vissouvanadin B., Le Roy S., Laurent C. (2015). Correlating conductivity and space charge measurements in multi-dielectrics under various electrical and thermal stresses. IEEE Trans. Dielectr. Electr. Insul..

[59] Le Roy S., Teyssedre G., Laurent C., Montanari G.C., Palmieri F. (2006). Description of charge transport in polyethylene using a fluid model with a constant mobility: Fitting model and experiments. J. Phys. D Appl. Phys..

[60] Chen L., Huan T.D., Ramprasad R. (2017). Electronic Structure of Polyethylene: Role of Chemical, Morphological and Interfacial Complexity. Sci. Rep..

[61] Tian F., Bu W., Shi L., Yang C., Wang Y., Lei Q. (2011). Theory of modified thermally stimulated current and direct determination of trap level distribution. J. Electrost..

[62] Rytöluoto I., Niittymäki M., Seri P., Naderiallaf H., Lahti K., Saarimäki E., Flyktman T., Paajanen M. (2022). Biaxially oriented silica–polypropylene nanocomposites for HVDC film capacitors: Morphology-dielectric property relationships, and critical evaluation of the current progress and limitations. J. Mater. Chem. A.

[63] Parsa S.E., Cañadas J.C., Diego J.A., Mudarra M., Sellarès J. (2022). Comparison of pulsed electroacoustic and thermally stimulated depolarization current measurements of thermally poled PET electrets. J. Electrost..

[64] Pei D., Shkel Y.M., Klingenberg D.J., Segal Z.I., Nishi Y., Shohet J.L. (2014). Measuring the volume charge in dielectric films using single frequency electro-acoustic waves. J. Mater. Res..

[65] van der Wal A., Nijhof R., Gaymans R.J. (1999). Polypropylene–rubber blends: 2. The effect of the rubber content on the deformation and impact behaviour. Polymer.

[66] Grein C., Gahleitner M. (2008). On the influence of nucleation on the toughness of iPP/EPR blends with different rubber molecular architectures. Express Polym. Lett..

